# *Drosophila* as a model to study the role of blood cells in inflammation, innate immunity and cancer

**DOI:** 10.3389/fcimb.2013.00113

**Published:** 2014-01-09

**Authors:** Lihui Wang, Ilias Kounatidis, Petros Ligoxygakis

**Affiliations:** Laboratory of Genes and Development, Department of Biochemistry, University of OxfordOxford, UK

**Keywords:** haemocytes, haematopoiesis, plasmatocyte, macrophage, innate immunity, tumor, inflammation

## Abstract

*Drosophila* has a primitive yet effective blood system with three types of haemocytes which function throughout different developmental stages and environmental stimuli. Haemocytes play essential roles in tissue modeling during embryogenesis and morphogenesis, and also in innate immunity. The open circulatory system of *Drosophila* makes haemocytes ideal signal mediators to cells and tissues in response to events such as infection and wounding. The application of recently developed and sophisticated genetic tools to the relatively simple genome of *Drosophila* has made the fly a popular system for modeling human tumorigensis and metastasis. *Drosophila* is now used for screening and investigation of genes implicated in human leukemia and also in modeling development of solid tumors. This second line of research offers promising opportunities to determine the seemingly conflicting roles of blood cells in tumor progression and invasion. This review provides an overview of the signaling pathways conserved in *Drosophila* during haematopoiesis, haemostasis, innate immunity, wound healing and inflammation. We also review the most recent progress in the use of *Drosophila* as a cancer research model with an emphasis on the roles haemocytes can play in various cancer models and in the links between inflammation and cancer.

## Introduction

*Drosophila* has undoubtedly been a powerful model organism for the study of nearly all essential and fundamental biological processes. What we have learned from the fruit fly has expanded our knowledge in life science at an unprecedented speed. This is in particular due to the recent availability of the complete annotated genome, a versatile array of genomic modifying techniques and powerful life imaging tools. Cellular and molecular mechanisms underlying many basic biological processes have been discovered to be highly conserved between *Drosophila* and mammals. For example, the Notch, Hedgehog (Hg) and Wingless (Wnt) pathways first identified in *Drosophila* embryogenesis and the Runt and Hippo signaling pathways conserved in the *Drosophila* haematopoiesis and tissue growth are also implicated in the progression of various human cancers (Geissler and Zach, 2012; Harvey et al., 2013). Indeed the past decade has witnessed a rapidly emerging trend for *Drosophila* to be used in modeling human tumor growth, progression, invasion and metastasis and as a test-bed for therapeutic discovery (reviews in Harris, 2005; Crozatier and Vincent, 2011; Miles et al., 2011; Hsu, 2012; Gonzalez, 2013).

Most forms of human cancers progress step by step from mutations in the oncogene, the tumor suppressor gene and signaling molecules and can eventually kill the host by spreading uncontrollable immortal growth of mutant malignant tissues into different organs. On the route to spread and invade, cancer cells can influence their microenvironment via the interaction with the infiltrated blood cells, gradually disabling the host immunosurvellience and finally breaking the stromal barrier to become invasive and metastatic (Dunn et al., 2004). It is at the metastatic stage that many lives would be claimed. Therefore the outcome from the tug of war in the tumor microenvironment between malignant cancerous cells that undergo constant somatic mutations and surrounding blood cells plays a vital role in the prevention and intervention of tumorigenesis. In addition, chronic inflammation has been well-documented as contributing to and promoting the initiation and progression of various cancers (Coussens and Werb, 2002; Mantovani et al., 2008; Aggarwal et al., 2009). It is now generally accepted that an inflammatory microenvironment is necessary for tumor progression and metastasis (Wu and Zhou, 2009; Grivennikov et al., 2010). Macrophages in particular have been reported to facilitate many aspects of this process in different cancers and also to intervene in the anti-cancer therapies (De Palma and Lewis, 2013; Lee et al., 2013b). Apart from the role of macrophages in cancer development, they have been for many years subjected to extensive research as the key player in inflammatory responses which accompany infection, tissue damage and wound healing (Mantovani et al., 2013; Novak and Koh, 2013). Therefore inflammation, immunity and cancer are inter-linked and any imbalance can result in serious health issues. Blood cells such as macrophages appear to be the link and have a crucial role in influencing and maintaining the equilibrium between protection (immunity and inflammation) and regeneration/tissue homeostasis (where cancer can be considered a malignant proliferative and invasive tissue). Animal models such as mice have revealed invaluable insights into the multi-step interaction of mammalian innate immunity with associated inflammatory responses in defining the cancer microenvironment. These innate immune responses can include the complement pathways (Ricklin and Lambris, 2013), pro-inflammatory cytokine and chemokine production (Sethi et al., 2012; Candido and Hagemann, 2013). However, the multi-layered interaction in the context of a generally slow progression of the human cancer has created fragmentary and controversial results in the mouse model and thus inevitably slows down our progress to understand the disease. *Drosophila*, on the contrary, as a simply-formed and genetically tractable multi-cellular organism, has been used to dissect processes of development (tissue homeostasis) and innate immunity with such precision that the time is now ripe for us to look into the active dialogs between these fundamental processes in the context of mammalian inflammation and cancer. *Drosophila* has a primitive open blood circulation system with only three types of blood cells or haemocytes circulating in the haemolymph during a fly's life span. The majority of the circulating haemocytes in the haemolymph are macrophage-like cells that engulf and degrade apoptotic cells and invading pathogens. Haemocytes perform vital roles through their contribution both to cellular and humoral immune responses in the fly. In combination with the currently well-developed *Drosophila* tumor models, the roles of haemocytes in tumor regression and/or progression can be explored and important clues can be obtained to understand further the inflammatory responses in relation to tumors; the focus can be directed more at the molecular and cellular level by use of sophisticated genetic manipulation and live imaging tools. Tian Xu and colleagues have done pioneering work in this direction by using a *Drosophila* tumor model to investigate the role of haemocytes in the tumor growth control during a systemic inflammatory response (Pastor-Pareja et al., 2008). In this review, we first give a brief overview of *Drosophila* haematopoiesis. This is followed by a discussion of the roles of haemocytes in *Drosophila* at different developmental stages. Next we consider how haemocytes function in tissue injury and wound healing. *Drosophila* leukemia model and interaction between immunity and tumorigenesis are also discussed. Finally, perspectives for possible future research opportunities in the interplay of inflammation, immunity and cancer revolving around the blood cells are discussed.

### *Drosophila* haematopoiesis

The blood system of *Drosophila* is rather primitive compared to the great complexity in vertebrates. The fruit fly does not have a vascular network to separate the blood cells from other tissues and organs and its internal organs are bathed in haemolymph. Meanwhile vertebrates have many different types of blood cells. Each type has evolved to perform specialized functions during million years of evolution. On the contrary only three major types of blood cells, collectively termed haemocytes, have been identified in the fruit fly and none of them has acquired the capability to undergo DNA rearrangement and somatic hypermutation to generate a vast repertoire for immunological memory in the B-and T-lymphocytes. Therefore *Drosophila* relies on a very simple system to fulfil basically all the roles that vertebrate blood cells can play. However there is extensive conservation in the molecular mechanisms of haematopoiesis in both *Drosophila* and mammals.

**Figure 1:**
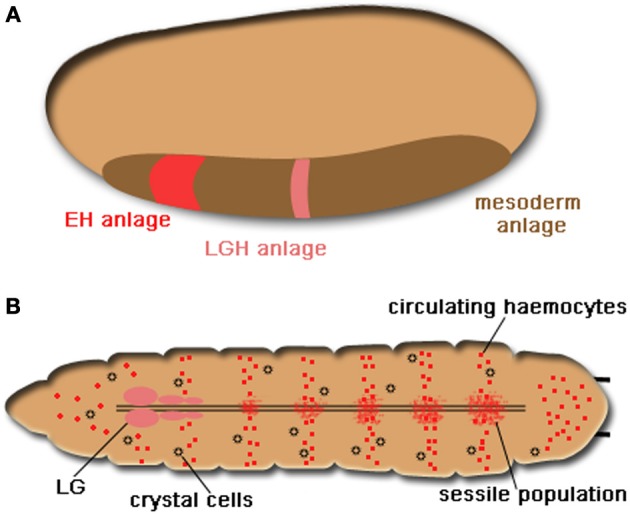
***Drosophila* haematopoiesis**. **(A)** The embryonic haematopoiesis. Two waves of haemocyte generation take place in *Drosophila*. During embryogenesis, haemocytes originate from the precephalic mesoderm as indicated in red in the region of mesoderm anlage (shown in brown), The pink region denotes the embryonic origin that will give rise to lymph gland haemocytes (LGH) in the larva. **(B)** Larval haematopoiesis. The LG (in pink) composes of the primary and secondary lobes and is located in the anterior end of the larva along the dorsal aorta. The sessile haemocyte population distributes diffusely along the segmental borders of the larva and consists of functional differentiated haemocytes and a few prohaemocytes with an embryonic origin (shown in the same red color). Until the end of the third star, circulating haemocytes including plasmatocytes and crystal cells (small black circles) are derived from the embryonic haemocytes.

**Figure 2:**
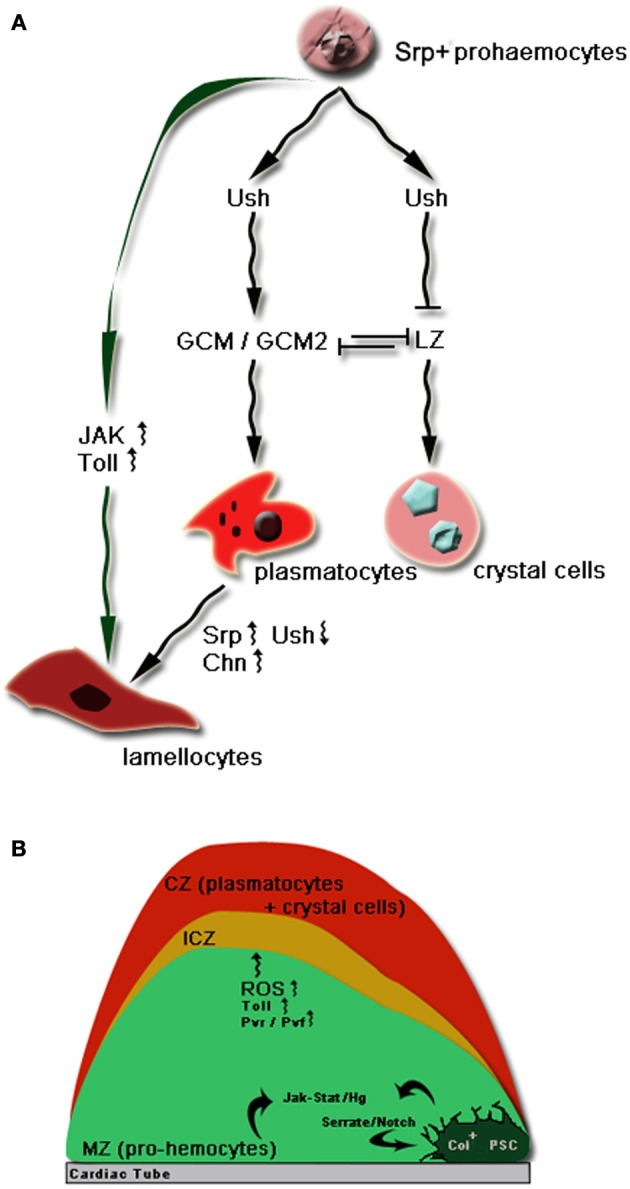
**(A)** The major transcriptional network in the lineage commitment of *Drosophila* prohaemocytes (Lebestky et al., 2000; Evans et al., 2003; Waltzer et al., 2010). Prohaemocytes express the early GATA transcription factor, Serpent (Srp) for haematopoietic development (Lebestky et al., 2000). The expression of Srp defines the haemocyte anlage from embryogenesis. Later on the Srp expressing prohaemocytes turn on the transcription of the *Drosophila* friend of GATA, U-shaped (Ush). Ush together with Srp activates the expression of another transcription factor Gilial cell missing (GCM) and its isoform GCM2. Activation of GCM/GCM2 turns on the expression of plasmocyte cell markers and thus commits prohaemocytes into plasmatocyte specification (Fossett et al., 2001). In a small population of prohaemocytes, the expression of a *Drosophila* Runx family member of transcription factors, Lozenge (Lz) antagonizes Ush and also inhibits the expression of Gcm/Gcm2. Cells expressing Lz adopt the crystal cell fate (Bataillé et al., 2005). Lamellocytes do not appear in normal circumstances but can be induced rapidly from the LG or sessile haemocytes by parasitic infection in the larva. Most recent lineage tracing studies have pointed out a direct differentiation of lamellocytes from plasmatocytes by the upregulation of Srp and Charlatan (Chn) and downregulation of Ush to suppress the plasmatocyte transcriptional profile (Stofanko et al., 2010). However direct differentiation from the LG prohaemocytes may also contribute to the total population of lamellocytes in particular by the over-activation of JAK/STAT and Toll signaling. **(B)** Compartments of the primary lobes of the LG and key signaling pathways in larval haemocyte specification (Krzemień et al., 2007). The primary lobes are the major sites for the larval haemocyte differentiation. The primary lobe can be divided into three major compartments: the Cortical Zone (CZ), the Medullary Zone (MZ) and the Posterior Signaling Center (PSC) (Jung et al., 2005). There is also a region that contains a population of intermediate haemocytes from undifferentiated prohaemocytes. This region is sometimes termed Intermediate Cortical Zone (ICZ). The stem cell-like fate of prohaemocytes is regulated by communication between the PSC cells and MZ via filopodia of the PSC cells. Activation of JAK/STAT and Hg signaling by the PSC cells maintains the undifferentiated status of prohaemocytes (Krzemień et al., 2007; Mandal et al., 2007). Meanwhile the transcription factor Collier (Col) expression defines PSC cell identity and is controlled by the Serrate/Notch signaling (Lebestky et al., 2003). Toll signaling is also important in the survival and proliferation of prohaemocytes while increased ROS level and Pvr/Pvf signaling can also contribute to the differentiation of prohaemocytes to plasmatocytes (Qiu et al., 1998; Brückner et al., 2004).

As in vertebrates, *Drosophila* haematopoiesis takes place in two phases: primitive haematopoiesis and definitive haematopoiesis (for more detailed review see Evans et al., 2003; Crozatier and Meister, 2007; Krzemien et al., 2010). Briefly, the site for primitive haematopoiesis resides in the precephalic mesoderm which gives rise to the early wave of haemocyte generation in the embryo (Figure 1A). At the end of embryogenesis, a specialized organ termed Lymph Gland (LG) originating from the lateral mesoderm starts to appear along the dorsal vessel and becomes fully mature during the first half of larval development (Figure 1B). Definite haematopoiesis initiates in the LG (Figures 1B, 2B) and generates terminally differentiated haemocytes at the onset of metamorphosis; the cells are released during the pupal stage with disintegration of the LG. After the disappearance of LG, no haematopoiesis will occur in the pupa or in the adult fly. Haemocytes persisting through the whole life stages of the fly therefore have either embryonic or larval lineage (Holz et al., 2003).

LG forms at the end of embryogenesis with a single pair of lobes called the anterior or primary lobes. Larval haematopoiesis takes place primarily in the primary lobes to temporally and spatially regulate the haemocyte differentiation. The primary lobes can be physically divided into three compartments (Figure 2B): the cortical and the medullary zones and the Posterior Signaling Center (PSC) (Jung et al., 2005). PSC is essential in controlling haemostasis in healthy larvae by a direct cell-cell communication via filopodia, thin cytoplasmic extensions (Krzemień et al., 2007; Mandal et al., 2007). This cellular contact provides a platform for the interplay of a network of key signaling pathways required for normal larval haematopoiesis (Figure 2A).

Based on different morphological features three major types of haemocytes can be identified throughout the life cycle of the fruit fly, namely, plasmatocytes, crystal cells and lamellocytes (Figure 2A) (Lanot et al., 2001; Hartenstein, 2006). Plasmatocytes are the dominating haemocyte population during all *Drosophila* developmental stages. They are macrophage-like cells that are primarily responsible for the removal of apoptotic debris, phagocytosis of invading microbes and repair of damaged tissues. Crystal cells are larger in size than plasmatocytes and are named from the paracystalline inclusions in the cytoplasm. The crystal cell inclusions are believed to contain large quantities of components involved in a process called melanisation involving a cascade of serine proteases leading to melanin synthesis (Jiravanichpaisal et al., 2006). Melanin is important to prevent haemolymph loss in wound sites, immobilize microbial pathogens and facilitate wound healing (see below). In addition, its free radical oxidative by-product can directly kill microorganisms. Although they constitute only a small proportion of 5% of the total population of *Drosophila* haemocytes in the embryo and larvae, crystal cells are major executioners in *Drosophila* innate immunity. Lamellocytes are physically distinctive from both plasmatocytes and crystal cells. They are flat and adhesive and are the largest haemocytes observed in *Drosophila* (Lanot et al., 2001). They do not appear in the embryo or in healthy larvae but can be induced quickly to differentiate from the LG or sessile population along the border of larval segments to engulf foreign particles larger than those that can be phagocytosized by plasmatocytes, such as the parasitoid eggs (Sorrentino et al., 2002; Lee et al., 2009). This process is termed encapsulation. Lamellocytes can also launch a melanisation cascade to kill the parasitic invaders with the aid of crystal cells and thus are essential in the *Drosophila* immunity against parasite infection (Krzemien et al., 2010).

The key players including primarily transcription factors and the corresponding molecular mechanisms are briefly summarized and illustrated in Figure 2.

### Haemocytes in immunity

In the wild, *Drosophila* feeds on rotting fruits and lives in a microorganism-enriched environment so fruit flies constantly face the danger of physical injury and gastrointestinal infection. The selection pressure from the hostile environment must be one of the driving forces for *Drosophila* to develop multi-layered defense responses so that it can survive and propagate. Not surprisingly the cellular and molecular mechanisms in the various facets of *Drosophila* innate immunity have been phylogenetically conserved. *Drosophila* can mount an array of cellular and humoral responses when challenged by pathogens such as bacteria, fungi, viruses and parasites. The cellular responses include direct engulfment of small objects such as the bacteria, as in phagocytosis, and encapsulation of larger objects such as parasitoid eggs. The humoral responses take place primarily in the haemolymph and three major events can occur during an immune challenge depending on the nature of the invading pathogen: (1) direct killing by Antimicrobial peptide (AMP) released into haemolymph from rapid de novo synthesis in the haemocyte and fat body; (2) direct killing by Hydrogen Peroxide (H_2_O_2_) or Nitric Oxide (NO) agents produced during melanisation; and (3) immobilization of opportunistic pathogens by blood coagulation at an open wound (for more in depth reviews see Lemaitre and Hoffmann, 2007; Kounatidis and Ligoxygakis, 2012). In this review, we focus on the roles of haemocytes in the *Drosophila* host defense and the key signaling pathways in orchestrating the different strategies deployed against a wide range of pathogens.

#### Molecular basis of haemocyte migration and motility in embryos

*Drosophila* haemocytes respond to numerous signals during development or following injury and/or infection (Wood and Jacinto, 2007). These signals can include migrating cues during embryogenesis, inflammatory and stress chemoattractants from the injury site and pathogen invasion in the haemolymph or in the tissue. During development, embryonic plasmatocytes are highly motile cells and they migrate from the precephalic mesoderm around stage 10 of the embryogenesis to start to disperse the entire embryo (Tepass et al., 1994). Haemocytes migrate along several invariant main routes throughout the embryo: toward the tail, along the ventral nerve cord, along the dorsal vessel and the gut primordium. The cell migration is guided by Pvr/Pvf signaling. The receptor tyrokinase Pvr is the homolog of the vertebrate Platelet-derived growth factor (PDGF) and Vascular endothelial growth factor (VEGF) receptors. The Pvr has three ligands: PDGF- and VEGF-related factor (Pvf)-1, Pvf-2 and Pvf-3. Haemocytes express Pvr and are attracted by Pvf2 and Pvf3 expressed in different tissues of the embryo. For example, the nerve cord expresses Pvf2 and Pvf3 spatially and temporally in its different compartments to attract haemocytes to move along the Central Nerve System (CNS) (Cho et al., 2002; Wood et al., 2006). The impressively fixed migrating patterns for haemocytes to populate the entire embryo from anterior to posterior and from dosal to ventral rely on sustained motility and cell polarity and highly organized cell shape to enable smooth and rapid movement along the tissue surface. By use of live confocal microscopy, plasmatocytes were discovered to move with large, polarized and actin-rich filopodia and lamellopodia during the embryonic migration (Wood et al., 2006). Toward the end of embryogenesis, these cytoplasmic protrusions become highly dynamic and continually extend and retract to survey the surrounding microenvironment. The Rho family small GTPase Rac, Rac1 and Rac2, function redundantly to control the lamellopodia formation and thus the successful dispersal of haemocytes in the embryo (Paladi and Tepass, 2004). A *Drosophila* PDZ guanine-nucleotide exchange factor (PDZ-GEF) Dizzy was also identified to be required for the embryonic haemocyte migration (Huelsmann et al., 2006). In the absence of Dizzy, the cytoplasmic protrusions are reduced in size and thus slow down the migration rate of the cells. Overexpression of Dizzy in haemocytes generates cells with abnormally extended protrusions. Dizzy is believed to act upstream of the Ras superfamily member of small GTPase Rap1 to regulate integrin dependent adhesion of haemocytes to the epithelia and to maintain their cellular “microspikes” throughout migration (Huelsmann et al., 2006). Siekhaus et al. discovered that during embryonic haemocyte migration *Drosophila* haemocytes invade an epithelial barrier as they move into the tail despite an open blood system (Siekhaus et al., 2010). A mutant of RhoL, another *Drosophila* GTPase homolog specifically expressed in haemocytes blocks this epithelia invasion but not other aspects of guided migration. RhoL interferes with Rap1mediated integrin adhesion by moving Rap1 away from a concentration in the cytoplasm to the leading edge during invasive migration. RhoL therefore functions as a regulator for integrin adhesion and Rap1 localization during the invasion. Inhibition of integrin-based adhesion is necessary to regulate the cadherin interactions that allow plasmatocytes to transmigrate from the head region, through the epithelium, to the posterior of the embryo. These findings revealed a striking similarity of the stepwise migratory process during *Drosophila* development with vertebrate immune cell transmigration during inflammation.

Apart from moving along fixed developmental migratory patterns, embryonic plasmatocytes respond to the epithelia wound by migrating rapidly to the site of injury. This process also shares many physiological relevancies with the vertebrate inflammation. Similar to the developmental migration, this deployment to the injury site also requires Rac-mediated lamellopodia formation (Stramer et al., 2005). Stramer and co-workers showed that Rho signaling is necessary for haemocytes to retract from sites of cell matrix and disengage from cell-cell contact. During the migration to the wound, CDC42 is required to maintain the plasmatocyte polarity. In contrast to the developmental dispersal of haemocytes in response to Pvr/Pvf cues, the chemotaxic signals from the injury site activate a different mechanism to mobilize plasmatocytes. This *Drosophila* inflammation induced cell migration depends on a phosphatidyl-inositol 3-kinase (PI3K) signaling which is also used by the mammalian neurophils in response to chemotaxic cues (Stramer et al., 2005). Therefore, it can be concluded that actin protrusion formation controlled by the Rac signaling in the cell motility is essential for plasmatocyte migration in these two different processes. Ena is another player identified recently to regulate the actin protrusions in the embryonic haemocyte (Tucker et al., 2011). Ena is the *Drosophila* homolog of Mena, member of the evolutionarily conserved Ena/VASP family of actin cytoskeletal regulators. Mena promotes metastasis and invasive motility of breast cancer cells *in vivo*. Tucker et al. found that Ena stimulates lamellipodial dynamics and positively regulates the number and length of filopodia. Overexpression of Ena in the haemocyte results in dramatic increase in the migration rate. One of the phenotypes can be also observed from overexpression of Mena in the mammalian fibroblast.

#### Postembryonic haemocyte migration

In the larva, overexpression of Rac in the haemocyte disrupts the sessile haemocytes population and causes a large migration of haemocytes into the circulation. Sessile haemocyte activation and mobilization require the Jun N-terminal kinase (JNK) Basket (Bsk) and Rac1. Bsk is also found to regulate the turnover of focal adhesions in the circulating haemocyte in the larva (Williams et al., 2006). These findings suggest that the Rho and JNK signaling are conserved, underlining their roles in the formation of cytoplasmic protrusions and actin focal adhesions for proper plasmatocyte mobility and migration to support their cellular roles in development and immunity. Two very recent studies on the postembryonic haemocyte migration have shed more light on the cellular dynamics and molecular basis of this process. An *ex vivo* culturing system using the primary larval or pre-pupal haemocytes has been developed to allow a real time analysis and manipulation to examine the roles of cytoskeleton dynamics in plasmatocyte migration (Sampson and Williams, 2012). From this system, it was found that larval circulating haemocytes are less motile than the pre-pupal haemocytes and thus unable to migrate. The extending and retracting rates of the protrusions appear dormant while the prepupal haemocytes have normal dynamic protrusions potentially required in the morphogenesis. The same study also reinforced the role of Rho family members: Rac1 and Rac2 and CDC42 to sustain the size of the filopodia and lamellopodia in the prepual haemocytes. Absence of these genes caused a static phenotype of pre-pual haemocytes similar to what was observed in haemocytes from third instar larvae. Though an *in vivo* assay still awaits to confirm these findings, the importance of Rho signaling in the actin cytoskeleton shaping has been strengthened in the ‘walking’ of the haemocyte along the extracellular matrix.

Another *in vivo* study based on MARCM (Mosaic Analysis with a Repressible Cell Marker System) investigated the integrin adhesion activation and maturation in the migration of the sessile haemocyte population in the late larval stage into pupal stage (Moreira et al., 2013). The *Drosophila* βPS integrin myosperoid and integrin containing adhesion regulators such as Rhea and Fermitin were found to be required in pupal haemocyte migration.

#### Cellular immunity mediated by plasmatocytes

Plasmatocytes represent around 90% of the total circulating haemocyte population in all developmental stages of *Drosophila*. They are professional macrophages which function as sentinels to maintain cell and tissue homeostasis and to recognize pathogen entry for subsequent immune reactions.

In the embryo, mature plasmatocytes function primarily as scavengers to remove apoptotic cell debris during embryogenesis. The clearance of apoptotic cell debris is dependent on scavenger receptors: CD36 homolog Croquemort (Franc et al., 1996), Draper (Manaka et al., 2004) and NimC4/Simu (Kurant et al., 2008). In the larval stage, recognition and rapid engulfment of invading microbes such as bacteria rely on the cell surface receptors Eater (Kocks et al., 2005) and NimC1 (Kurucz et al., 2007) and in the adult fly on Draper also (Cuttell et al., 2008). Loss of function of mutants in those receptors results in functional deficiency of phagocytosis of Gram-positive bacteria such as *Staphylococcus. aureus* and Gram-negative bacteria such as *Escherichia coli*. Eater, NimC1, NimC4 and Draper together with CED in *C. elegans* belong to a large protein family conserved across the metazoan animal kingdom including other insects such as *Anopheles* and humans (Kurucz et al., 2007). Epidermal Growth Factor (EGF)-like repeats are abundantly found in the extracellular domains of these bacterial phagocytosis receptors and there is evidence to show the direct interaction and binding of the EGF-like repeats with bacteria in Eater (Kocks et al., 2005) and Draper (Hashimoto et al., 2009). These interesting findings suggest that proteins with GF-like repeats may play an evolutionary conserved role in phagocytosis in the entire animal kingdom (Table 1).

**Table 1:** **Key genes involved in innate immunity, inflammatory responses and wound healing in *Drosophila* haemocytes**.

**Blood cells**	**Innate immune response**	**Key genes**
Plasmatocytes	Phagocytosis / AMP production	Croquemort (Crq)
		Draper
		NimC4/Sim
		Eater
		NimC1
		PGPR-LC/PGRP-LE
		Dscam
	Blood coagulation	Hemolectin (hml)
		Transglutaminase
		(TF)
	Inflammation / Wound healing	Eiger
		Psidin
		Spatzle
	Cytokine-like	Hayan
		UPd3
		Rac1/Rac2
		Dizzy
		Zir
	Cytoskeleton modulation	RhoL
		Rap1
		Cdc42
		Ena
Crystal cells	Wound healing / Melanisation	Srp7
		DoxA1
		CG8193
Lamellocytes	Parasite infection	DoxA3
		Charlatan

Another receptor that binds directly microorganisms and participates in phagocytosis is Dscam, which is a member of the Ig superfamily with an essential function in neuron interconnection. By alternative splicing as many as 18,000 isoforms can be theoretically generated in the haemocyte and fat body (Watson et al., 2005). Haemocyte-specific Dscam silencing reduces the phagocytic uptake of bacteria. The existence of a potential extensive repertoire of thousands of Ig-domain-containing proteins in the recognition of a variety of pathogens in *Drosophila* and other invertebrates has opened up a new avenue in exploring the possibility of an “adaptive” immunity across animal plyla that have been considered as having only innate responses (Watson et al., 2005; Schmucker and Chen, 2009).

#### Haemocyte-mediated humoral response

In response to pathogens that manage to gain entry into the haemocoel *Drosophila* can mount a robust systemic immune humoral response. The hallmark of this response is the rapid synthesis of a broad spectrum of AMPs against bacteria and fungi both in the haemocyte and in the fat body. AMPs are secreted into the haemolymph and directly kill the microbes at an optimal concentration. Although the fat body—the *Drosophila* equivalent of mammalian liver—is the prominent site of AMP synthesis, plasmatocytes play important roles in triggering the AMP production as haemocyte ablation can abolish the AMP expression in larval fat body (Shia et al., 2009). The roles plasmatocytes can play in the systemic immune response can be achieved by signaling between the site of infection and the fat body or by degradation of invading pathogens. To date, a haemocyte-released cytokine, Unpaired-3 (Upd-3) has been proposed to activate the JAK/STAT pathway in response to septic injury by binding to the fat body Domeless (Dom) receptor (Agaisse et al., 2003). Nevertheless the precise role of this pathway and its overall contribution to the host defense remains to be established. Spätzle is another cytokine secreted by haemocytes, processed by a serine protease cascade in the haemolymph and required for the Toll signaling pathway controlled AMP synthesis in the fat body (Shia et al., 2009). The Toll signaling has been well characterized in the Dorsal-Ventral patterning during embryogenesis and AMP production against mainly fungi, Gram positive bacteria, *Pseudomonas aeruginosa* (a Gram negative bacterium) and also stress/danger signals (reviews in Valanne et al., 2011; Kounatidis and Ligoxygakis, 2012). The core Toll signaling event is the degradation of *Drosophila* NFκB Inhibitor homolog Cactus followed by the activation and translocation of the *Drosophila* NFκB transcription factors Dorsal or Dorsal related immunity factor (Dif) into the nucleus. Dorsal and Dif are homologs of mammalian p50 and p65. Apart from these secreted cytokines, a cytoplasmic lysosomal protein called Psidin has been found to be the link between the haemocyte phagocytosis and AMP activation in the fat body in the larval immune response (Brennan et al., 2007). Psidin is required both for the phagocytic degradation of internalized bacteria and for the induction of one of the AMPs, Defensin, in the fat body. This interesting finding suggests a likely “antigen” presentation mechanism of the haemoctye to the fat body for the activation of AMP. Contrary to these findings, plasmatocyte ablation does not affect the antimicrobial responses upon systemic infection in the adult fly (Charroux and Royet, 2009; Defaye et al., 2009). This might suggest that tissue specific humoral responses, such as local expression of AMP and cytokines independent of haemocytes in the gut or in the trachea, play dominant roles in the adult immunity against pathogens.

#### AMP production in haemocytes

AMP production plays a vital role throughout the life cycle of *Drosophila*. Many tissues that have direct contact with the microorganisms such as the trachea, the gut and malpighian tubules have the capability to synthesize AMP and kill the microbes locally and efficiently. For microbes that manage to gain entry into the circulation via an open wound or the digestive or reproductive tracts, the fly can mount a systemic humoral response to produce large amounts of AMP from mainly the fat body into the haemolymph. Although haemocytes are not the major organ in the fly for systemic AMP production, the signaling pathways in control of AMP synthesis are activated in haemocytes like in the other tissues during a concerted immune response, in particular in the embryonic haemocytes (reviewed by Lemaitre and Hoffmann, 2007; Kounatidis and Ligoxygakis, 2012).

#### Melanisation

Melanisation in arthropods is generally believed to play an important and central role in arthropod defense reactions such as wound healing, encapsulation, microbe immobilization and the production of toxic intermediates that are speculated to kill invading microorganisms (Cerenius and Söderhäll, 2004). As described briefly above, crystal cells are the major haemocytes responsible for the melanisation reaction in the larva. Melanisation can be immediately induced at the site of cuticular injury or on the surface of parasites invading the haemocoel. It involves formation of black pigmentations resulting from *de novo* synthesis and deposition of melanin. Prophenoloxidase (PPO) is the enzyme required in melanisation to catalyze the oxidation of mono- and di-phenols to ortho-quinones, which polymerize into melanin. PPO in normal physical conditions is enzymatically inert. A serine protease known as prophenoloxidase activating enzyme (PPAE) acts upstream to cleave and turn PPO into active phenoloxidase (PO). Like PPO, PPAE also exists as an inactive zymogen and it is processed by a tightly regulated serine protease cascade in a step wise way into enzymatically functional form leading to the final melanin formation. As in other invertebrates, a recent *in vitro* study on the *Drosophila* PPOs suggested a direct binding of PPOs to bacteria and fungi which might play a role to initiate their activation (Yang et al., 2013). The *Drosophila* genome encodes three PPOs: DoxA1, DoxA3 and CG8193 (Irving et al., 2005). Crystal cells express DoxA1 and CG8193 while lamellocytes express exclusively DoxA3, a strong indication that Dox3 participates in the encapsulation that accompanies melanisation. Melanisation is diminished in the *domino* mutant that lacks haemocytes (Braun et al., 1998) and in the *Black cells* (Bc) mutant with aberrant crystal cells (Rizki et al., 1980; Corbo and Levine, 1996) and the Lz knockout, which is devoid of crystal cells (Peeples et al., 1969). One serine protease Sp7 has been reported to be involved in PPO activation and expressed also in the crystal cells (Castillejo-López and Häcker, 2005). In the absence of crystal cells in the adult fly, melanisation perhaps relies on the activation of proteolytic cascades in the haemolyph including PPAE, PPO and serine proteases. The cascades are tightly regulated by serine protease inhibitors in the haemolymph such as Serpin27 A to restrict the reaction to the site of injury and to prevent the spread of systemic melanisation (De Gregorio et al., 2002; Ligoxygakis et al., 2002). Two serine proteases MP1 and MP2 are reported to activate the cascade in response to different microbial changes (Tang et al., 2006). This pathogen-specific activation of melanisation can be attributed to PGLP-LC and PGRP-LE expressed both by haemocytes and the fat body (Takehana et al., 2004; Schmidt et al., 2008). However the connection between other types of pathogen receptors (for example, Gram-positive bacteria and fungi etc.) has not been linked to melanisation triggering. To date only one PPAE has been identified in the melanisation cascade of the adult fly (Leclerc et al., 2006).

#### Encapsulation

Lamellocytes are the major executioner of encapsulation during parasite infection in the *Drosophila* larva. Encapsulation involves three key steps with coordinated actions from both plasmatocytes and lamellocytes. Firstly circulating plasmatocytes sense and recognize the entry of parasitoid eggs in the haemocoel and attach to the egg chorion. Secondly a massive proliferation and differentiation of sessile compartments and of haemocytes in the LG to lamellocytes is induced via unknown signaling molecules within a few hours to appear in the circulation where the lamellocytes form a multi-layered capsule around the eggs. Eventually the lamellocytes, like the crystal cells, release their cellular content such as PPO to activate the melanisation process and kill the parasites, possibly by the cytotoxic by products from the localized melanisation reaction (Nappi et al., 1995). To date genes that have been reported to play a role in encapsulation process are involved primarily in cell-cell interaction such as αPS4/βPSintegrins (myospheroid) (Irving et al., 2005; Wertheim et al., 2005) and in cytoskeleton remodeling for motility and migration, such as RhoGTPase protein family member Rac1(Williams et al., 2006), Rac2 and CDC42 (Williams et al., 2005). Rac2 and CDC42 are activated by the *Drosophila* homolog of Rho guanine nucleotide exchange factor (RhoGEF), Zir (Sampson and Williams, 2012; Sampson et al., 2012). Rac1 and Rac2 function in a non-redundant manner. Interaction between Rac1and myospheroid has recently been reported to be required in the directed localization of β-integrin on the cell surface of lamellocytes in response to parasitoid eggs (Xavier and Williams, 2011). Rac1 requires the JNK pathway component Bsk to regulate the formation of actin- and focal adhesion kinase (FAK)-rich placodes in haemocyte migration and both are required for the proper encapsulation of wasp eggs (Williams et al., 2006). A recent screen to target genes involved in the cell adhesion and shape change not only strengthened the previous findings but also discovered more conserved components in these cellular processes that participate in the encapsulation reaction, for example the extracellular matrix proteins (ECM) (Howell et al., 2012). Loss of function of ECM components results in failure to encapsulate. In correlation with the previous discovery on the encapsulation of mechanically damaged self-tissue, it is plausible that exposure of ECM by foreign particle intrusion or deposition of ECM on the eggs can be the initiative signal in encapsulation (Rizki and Rizki, 1980; Howell et al., 2012). Genome-wide analysis of the transcriptional profiles in haemocytes after parasitoid infection has offered many interesting and promising candidate genes that are differentially regulated in the encapsulation reaction (Wertheim et al., 2005). This study also reinforced the importance of the Toll and JAK/STAT signaling pathways in the differentiation and proliferation of lamellocytes in the LG (Sorrentino et al., 2004). In addition, the haemocyte-specific transmembrane protein Hemese has been reported to play a modulatory role to keep lamellocyte proliferation in check from overacting during parasitoid egg infection (Kurucz et al., 2003). Despite these findings, the molecular nature of the signals sent from plasmatocytes for lamellocyte differentiation in the LG remains elusive. It has been proposed that a signal delivered to the PSC initiates lamellocyte differentiation as the PSC-restricted expression of Collier (Col), the *Drosophila* homolog of human Early B cell factor, is required upon parasite invasion (Crozatier et al., 2004).

#### Blood coagulation

Haemocytes have essential roles in blood coagulation, not only to maintain haemostasis but also to defend against pathogens. It has been found that Hemolectin (Hml) expressed mainly by plasmatocytes is required in blood coagulation. Blood coagulation led by plasmatocytes is independent of both melanin production and phenoloxidase activity, which is also part of the wound healing process (Goto et al., 2003). By proteomics and pull out analysis, important components of the blood clot have been isolated and subjected to detailed genetic and cellular investigations (Karlsson et al., 2004; Scherfer et al., 2004). Among these the best-characterized clotting factors are *Drosophila* Transglutaminase (TF) and Fondue. *Drosophila* TF is the only mammalian blood coagulation factor homolog (Factor XIIIa) found in the fly and uses Fondue as its substrate to form the blood clot. Unlike *hemolectin* (*hml*) mutants shown to affect only coagulation (Goto et al., 2003), ubiquitous silencing of *fondue* also results in cuticle defects in the pupa as well as in the clot forming in larvae (Scherfer et al., 2006). Hml is expressed mainly by plasmatocytes and contains domains found in coagulation factors (Goto et al., 2001, 2003). It is suggested that TF/Fondue acts more actively in cross-linking of fibers formed by Hml, reacting promptly to bleeding and injury (Scherfer et al., 2006; Lindgren et al., 2008). Interestingly TF is most likely expressed in haemocytes (Johansson et al., 2005) while Fondue is expressed in the fat body under control of the Toll signaling pathway (Scherfer et al., 2006). This strongly suggests that cellular and humoral factors are required in blood coagulation with contributions from both haemocytes and the fat body. Therefore, the lack of a signal sequence in TF gene (like PPO, another enzyme expressed in haemocytes) suggests that its release from haemocytes may be a key step in the initiation of coagulation. Most recently Wang et al. proposed a conserved innate immune mechanism based on TF's ability to use a potential microbial surface substrate to sequestrate and immobilize bacteria to the clot formed in blood coagulation (Wang et al., 2010).This interesting piece of work provides direct evidence for the blood coagulation factor to directly bind to microbes in the process of blood clot formation.

### The *Drosophila* “inflammatory response”

All organisms have developed various mechanisms to maintain structural and physiological integrity in response both to external injury and to internal disruption. Wound healing, tissue repair and regeneration are essential processes for multi-cellular organisms to survive and proliferate against constant environmental or physical assaults. Therefore it is highly feasible to hypothesize that wound healing is an ancient process that evolved before the divergence of insects and mammals and this view can be supported by evidence from extensive research conducted in various model organisms on the pathways underlying this basic process.

Wound healing depends on complex molecular and cellular networks involving different types of cells and tissues. This complexity increases with the basic mechanisms varying in a tissue-specific, developmental stage dependent, and damage related manner. For example, in fruit fly embryos wound healing occurs rapidly via actin cable assembly and filopodial extension by cells at the wound margin, and proceeds without blood clot formation (Kiehart et al., 2000; Wood et al., 2002). Again, despite the substantial structural differences between *Drosophila* and mammalian epidermis, embryonic wound healing in mammalian embryos appears to be similar to that in *Drosophila*. It is also a rapid process involving actin cable formation without apparent haemostatic or inflammatory response (Martin and Lewis, 1992).

#### Haemocytes in embryonic wound healing

Although blood clots are not formed and required during the embryonic wound healing, microarray analysis comparing the transcriptional profile of wild type and haemocyte-absent embryos still revealed interesting haemocyte signature genes involved specifically in wound healing. These include phospholipase A2 conserved also in the mammalian inflammatory response (Stramer et al., 2008). From this study, a *Drosophila* ortholog of a novel mouse inflammatory-responsive gene Growth Arrest and DNA Damage-inducible gene 45 (GADD45) (Takekawa and Saito, 1998) was found to be induced in the damaged epidermal cells. This finding reinforces the idea that inflammatory responses are ancient processes for organisms to respond to danger signals. JNK signaling has also been reported to be essential in the epithelial wound healing in the embryo (Rämet et al., 2002; Wood et al., 2002). The earliest signal that triggers the haemocyte attraction to a wound in embryos has been recently identified as the calcium wave from the damaged epithelial cells following immediate laser wounding. Blocking this calcium flash inhibits H_2_O_2_ synthesis which relies on the activation of an NAPDH oxidase, DUOX (Razzell et al., 2013). In response to H_2_O_2_ that transiently outcompetes developmental migrating cues, haemocytes are quickly recruited to the injury site in the embryo (Moreira et al., 2010). The establishment of calcium flux-induced H_2_O_2_ production in the inflammatory response associated with wound healing from *Drosophila* will certainly give more insights into the signaling events taking place in wound induced inflammation in mammals.

#### Haemocytes in tissue injury and wound repair in the larva

The mammalian epithelial tissue can summon a set of humoral and cellular reactions lasting from days to months in response to tissue damage until the damage cap is properly closed and the injured cells or tissues are removed and replaced. The reactions typically include the rapid formation of a blood clot at the injury site and recruitment of inflammatory blood cells followed by spreading of the damaged epithelium across the wound gap to restore tissue integrity (Of and Healing, 1999; Singer and Clark, 1999). Likewise, *Drosophila* larval wound healing shares many similarities to postembryonic wound healing in mammals. By developing an aseptic puncture wounding in the third instar larva in combination with *in vivo* life imaging, Galko and Krasnow (2004) have established a system to study the process of *Drosophila* postembryonic wound healing. They have characterized the wound healing process by three key stages: (1) primary clot formation during blood coagulation: larvae bleed following the puncture wounding and the primary clot forms in the wound gap; (2) scab and syncytium formation: the primary clot is further cross-linked and hardened by melanisation to form a scab while epidermal cells surrounding the primary clot migrate toward it and then fuse to form a syncytium; and (3) central syncytium formation: more epidermal cells are attracted to the initial synticium and a larger central syncytium forms. JNK pathway is activated in the epidermal cells of the syncytium in a gradient manner to emanate signals for the epidermal cells to move along or through the wound clot to rebuild a continuous epithelium with its basal lamina and apical cuticle lining. Crystal cells are the haemocyte required in the formation of the scab. The scab stabilizes the wound site, establishes a physical barrier to the external microbes, prevents the over-activation of JNK pathway which can result in chronic wounding and provides a scaffold for re-epithelialisation. Scab forming and wound closure are controlled by independent genetic and signaling pathways as re-epithelialisation can still be activated at the wound gap though it never heals in the absence of a scab (Galko and Krasnow, 2004). Presumably, multiple signals must be produced to spatiotemporally co-ordinate this dynamic flow of cellular events involving crystal cells, epidermal cells and also plasmatocytes to remove cell debris for tissue remodeling in addition to phagocytosis of invading microbial pathogens from the open wound. For example: the signals from damaged sites to initiate blood coagulation as discussed in the previous section are still to be identified. What also remains unknown at present is the signal to attract crystal cells to the primary clot, the signals during the formation of a scab (perhaps from crystal cells or plasmacytes) to negatively modulate the JNK activity and the signal to recruit plasmatocytes to the wound site. These signals may be able to behave like mammalian chemokines or cytokines and possess distinct characteristics in terms of the range and the different signaling pathways they can activate. In addition, there could also be mitogenic signals from apoptotic cells to stimulate cell migration and regeneration (Bergmann and Steller, 2010). Although the identity of these invertebrate inflammatory signaling molecules remains largely unknown, in combination with studies on other arthropods the interconnection between inflammatory responses and wound healing seems to be phylogenetically conserved (Theopold et al., 2004; Eleftherianos and Revenis, 2011). Recent studies have begun to reveal the molecular identities of some of these signals. A blood borne Pvf1 ligand has been found to be expressed by epidermal cells at the wounding edge and to function in an autocrine manner to activate the motility of epidermal cells in wound closure (Wu et al., 2009). In a study by mutant screening using crystal cell rupture and melanisation as the readout, Bidla et al. reported that the rapid rupture of crystal cells and subsequent local melanisation in the clot at injury depended on the JNK pathway and on Eiger, the *Drosophila* homolog of tumor necrosis factor (Bidla et al., 2007).The most interesting finding is that endogenous signals such as Eiger released from crystal cells and plasmatocytes undergoing apoptosis followed by secondary necrosis can function independently of microbial elicitors in triggering the PPO activation, which can support the idea that endogenously induced ‘death signals’ initiate inflammatory and repair responses. Nevertheless, the molecular nature of the signal to initiate JNK pathway by the mechanical stress for the crystal cells to rupture still remains to be discovered to date.

In a study to reveal the genetic and molecular networks in control of systemic wound response after physical wounding in *Drosophila* larvae and adult flies, Nam et al. reported that a redox signal released from proPPO activation via the blood borne serine protease Hayan is required for the downstream activation of JNK signaling to protect remote internal tissues from systemic wound response induced by local physical trauma (Nam et al., 2012). Forced expression of Hayan in the haemocyte, but not in other tissues, rescued the wound induced mortality in the *hayan* loss of function mutant suggesting that a redox dependent mechanism communicates between circulating haemocytes (most probably plasmatocytes) and the remote internal tissues in a process similar to systemic inflammation in mammals.

The cellular and genetic basis of wound healing has recently been studied in detail by using a *Drosophila* embryo laser wounding model and it is revealed not surprisingly that a coordinated process exists involving myosin, E-cadherin, Echinoid, the plasma membrane, microtubules and the CDC42 small GTPase which respond dynamically during wound repair (Abreu-Blanco et al., 2011, 2012). The wound healing mechanism in *Drosophila* larvae was also explored by the development of a targeted large scale *in vivo* RNAi screen in the larval epidermis. Likewise, in the embryo, components in the JNK pathway and genes involved in the remodeling of actin cytoskeleton also actively participate in the larval wound healing (Lesch et al., 2010). Key genes in the haemocyte mediated innate immunity and in the *Drosophila* inflammatory response are summarized in Table 1. For recent reviews on the topic of wound healing, (see Belacortu and Paricio, 2011; Ríos-Barrera and Riesgo-Escovar, 2013).

#### Haemocytes in infection induced inflammation

The last decade has witnessed a rapid growth of research in gut immunity in *Drosophila* (reviews in Royet, 2011; Kounatidis and Ligoxygakis, 2012). In 2009 a genome-wide RNAi screen revealed a large numbers of genes in both haemocytes and the fat body to be regulated following intestinal *Serratia macescens* infection (Cronin et al., 2009). By ontology enrichment analysis, this study found a strong enrichment of genes in haemocytes implicated in processes including phagocytosis, responses to external stimuli and vesicle trafficking. The critical role of JAK/STAT signaling in the gut immunity was reinforced to function through regulation of intestinal stem cell proliferation which controls the gut epithelial cell haemostasis. Though potential signaling pathways were not the focus of this genome-wide analysis, the large number of genes to be either upregulated or downregulated in haemocytes suggests important modulatory roles that haemocytes can play in organ to organ communication in response to internal infection or inflammation. Recently, Juang and colleagues found that intestinal ROS signal triggers a systemic AMP expression in the fat body following oral feeding of Ecc15 (*Erwinia carotovaro* subsp.) to larvae. The ROS stress in the gut induces NO expression and transduces the signal to haemocytes by a NO dependent pathway (Wu et al., 2012). NO-dependent signaling mediated by haemocytes has been also observed following gastrointestinal infection by *Candida albicans* (Glittenberg et al., 2011). In the absence of haemocytes, the AMP production was greatly reduced but not completely abolished in the fat body. Though ROS has long been recognized to be involved in the initiation of inflammatory bowel diseases in humans (Rezaie et al., 2007), this recent finding from *Drosophila* can offer further insight into the potential role of macrophages in triggering a systemic inflammation during inflammatory bowel diseases. In addition, gut-associated macrophage-like cells were also found in the larval gut and their number was regulated by the PI3K signaling pathway (Zaidman-Rémy et al., 2012). This situation closely resembles the mouse colitis model (for review see Lin and Hackam, 2011). Taken together, research in *Drosophila* has revealed many similarities in the important signaling roles macrophage-like blood cells can fulfil during either local tissue-specific or systemic inflammation following internal infection. On the other hand, more recently, Panayidou and Apidianakis have given a comprehensive review on using *Drosophila* as the model to study interlinking mechanisms underlying intestinal cell proliferation, differentiation and maintenance during bacterial infection and intestinal stress. The authors therefore proposed a regenerative inflammation phenomenon independent of haemocytes conserved between *Drosophila* and mammals in cancer progression (Panayidou and Apidianakis, 2013).

### Cancer and immunity in *Drosophila*

#### *Drosophila* as the model to study haematopoiesis and its associated leukemia

In adult mammals such as humans and mice, bone marrow is the organ that houses haematopoietic stem cells (HSC) which give rise to both the myeloid and lymphoid lineage. Mammalian HSC possesses the ability to self-renew and the pluripotency to differentiate into a great variety of blood cells in response to signals from its microenvironment, which has been termed the HSC niche (review in Wang and Wagers, 2011). The HSC niche has been a subject for vigorous research since the concept has been fully accepted (Wang and Wagers, 2011; Lensch, 2012). The dynamic communication between HSC and its niche has been shown to be fundamental in the control and regulation of haematopoietic process in vertebrates. Any dysfunction in the genetic and cellular mechanism underlying the HSC and niche interaction can result in blood borne cancers such as AML (Oh and Humphries, 2012). However, the structural and cellular complexity of the bone marrow niche has hindered the progress of fully understanding the basic genetic and molecular events fundamental to the haematopoietic process and their application in potential human diseases. The *Drosophila* larval PSC as discussed previously functions as a primitive niche to instruct the different fate that the prohaemocyte would adopt or to help the prohamemocyte to maintain its stem cell status (Crozatier and Meister, 2007). The LG primary lobes represent a very simplified HSC and niche model compared to its mammalian counterpart (Mandal et al., 2004). Although there are obvious restraints such as the limited number of differentiated blood cell types and the complete absence of lymphocytes, this simplicity can further our understanding of the basic signaling and cellular communication mechanisms involved in particular between the HSC and its microenvironment (review in Crozatier and Vincent, 2011).

#### Signaling pathways in *Drosophila* haematopoiesis and tumorigenesis

Research in *Drosophila* haematopoiesis has revealed a number of pathways in control of prohaemocyte proliferation and differentiation. Overexpression of the *Drosophila* JAK gene *hop*^*Tum*−*1*^ causes proliferation of prohaemocytes and leads to melanotic tumor formation in the LG (Harrison et al., 1995). This discovery preceded the demonstration that mutated constitutive activation of JAK/STAT signaling could result in human leukemia (Lacronique et al., 1997). Compared to the vertebrate system, the *Drosophila* JAK/STAT pathway is much simplified and shows nearly complete absence of genetic redundancy. The *Drosophila* genome encodes only three upstream ligands of JAK/STAT pathway Unpaired (Upd1-3) while the mammalian JAK/STAT can be activated by a large group of cytokines and growth factors. Dom is the only transmembrane receptor upstream of one JAK kinase (Hop) and the one STAT transcription factor (STAT92E). Therefore the misexpression of a dominant-active form of STAT92E can also promote tumorigenesis in the eye of the *Drosophila* adult flies and melanotic tumor formation in the larva (Ekas et al., 2010). A systematic genome-wide RNAi screening for genes required for JAK/STAT pathway activity in cultured *Drosophila* haemocyte-like cells also identified interacting genes that can function as suppressors of leukemia-like blood cell tumors in humans (Müller et al., 2005). Increasing evidence from clinical research in human AML has pinpointed a role for JAK/STAT signaling pathway to be implicated in AML pathogenesis. In particular an activating mutation on the human JAK2 has been discovered to be responsible for various forms of AML (Lee et al., 2013a; Vainchenker and Constantinescu, 2013).

Apart from the JAK/STAT signaling pathway, the Notch, Hg, Wnt, and JNK pathways have all been identified as regulators of prohaemocyte fate (Mandal et al., 2007; Owusu-Ansah and Banerjee, 2009; Sinenko et al., 2009). Hg signaling in the PSC has been identified to maintain the undifferentiated fate of prohaemocytes in the larval medullary zone of the primary lobes in the LG (Mandal et al., 2007). In a mouse B cell lymphoma model, Hg signaling from the stromal cells was also shown to provide an important survival signal for B- and plasma-cell malignancies *in vitro* and *in vivo* (Dierks et al., 2007).The Hg signaling pathway was also discovered to be required in the maintenance of cancer stem cells of chronic myeloid leukemia (CML) (Zhao et al., 2009). Loss of Smoothened (Smo) the downstream transmembrane G protein coupled receptor in the JAK/STAT pathway causes depletion of CML stem cells whereas constitutively active Smo augments CML stem cell number and accelerates the disease. These studies are reminiscent of Hg signaling implicated in the communication between PSC niche and the prohaemocyte in *Drosophila* haematopoiesis.

Notch signaling pathway has been well conserved in vertebrate haematopoiesis, in particular in lymphoid cell commitment (Radtke et al., 2005; Tanigaki and Honjo, 2007). In *Drosophila* LG, the Serrate-mediated Notch signaling from the PSC is required to maintain normal levels of Col transcription and thus PSC cell identity (Krzemień et al., 2007). In addition, a non-canonical and ligand-independent activation of Notch signaling has also been reported to determine crystal cell fate in the LG. The *Drosophila* ortholog of mammalian hypoxia-inducible factor–a (HIF-a), Sima, activates full length Notch receptor under conditions of normal oxygen availability and commits the prohemocyte to the crystal cell lineage (Mukherjee et al., 2011). In fact, the first human Notch was originally identified from human Acute lymphoblastic leukemia (ALL) and since then Notch signaling has been discovered to be involved in many forms of human leukemia (review in Pancewicz and Nicot, 2011).

Wnt signaling has been shown to promote proliferation of prohaemocytes and prevent differentiation at the same time by controlling the PSC niche (Sinenko et al., 2009). In addition, Wnt signaling also positively regulates the proliferation and maintenance of PSC cells while inhibition of Wnt signaling results in fewer PSC cells than observed in control flies. Likewise in vertebrates, Wnt signaling has been reported to play important roles in the HSC homeostasis and maintenance of its microenvironment for self renewal (for the most recent review see Seke Etet et al., 2013) and thus has been implicated in many forms of haematologic malignancy such as AML (Gandillet et al., 2011) and CML (Nagao et al., 2011). To elucidate the controversial role of reactive oxygen species (ROS) in the haematopoietic system, work done by Owusu-Ansah and Banerjee set out to make use of the *Drosophila* haematopoietic model to study levels of ROS in the *in vivo* proliferation and differentiation of prohaemocytes. It was found that increased levels of ROS promote the differentiation of prohaemocytes while inhibition of the ROS level delays the differentiation of prohaemocytes into mature haemocytes. Interestingly, through a downstream signaling pathway that involves JNK and FoxO activation as well as Polycomb downregulation, increasing the haematopoietic progenitor ROS beyond their basal level triggers premature differentiation of prohaemocytes into all three mature haemocytes found in *Drosophila*. Therefore a moderately high level of ROS can be the developmental signal for the population of haemocyte progenitor to commit to lineage differentiation (Owusu-Ansah and Banerjee, 2009) (Figure 2B). In mammals, higher ROS level was also observed during the common myeloid progenitor differentiation in response to oxidative stress (Tothova et al., 2007). A recent study by Dragojlovic-Munther and Martinez-Agosto demonstrated that the tumor suppressors TSC and PTEN also have important roles in controlling blood progenitor proliferation through a common TOR- and 4EBP-dependent pathway in the LG. Loss of function of *Tsc2* or *Pten* in prohaemocytes increases TOR signaling and causes overgrowth of the LG by haemocyte hyper-proliferation accompanied by a higher level of ROS. This study illustrates further how TSC and PTEN influence TOR function in response to physical stress such as starvation, hypoxia or increased ROS level during infection (Dragojlovic-Munther and Martinez-Agosto, 2012). Interestingly, the PTEN/mTOR signaling pathway has indeed been the therapeutic target in the treatment of human leukemia (Martelli et al., 2011).

Collectively these studies demonstrate the strength of *Drosophila* as an excellent model to study the HSC and its microenvironment interaction and shed light on the potential for therapeutic prevention of various hematological malignancies by dissecting its underlining genetic and cellular mechanisms.

#### *Drosophila* human leukemia model

Apart from the elucidation of basic molecular signaling pathways in the HSC and its niche interaction, *Drosophila* can also be used directly to model human leukemia. In addition to overactivation of JAK/STAT signaling, human AMLs can result from the chromosomal translocation of the transcription factor AML1, a RUNX domain protein, to form a protein fusion product with ETO (Hatlen et al., 2012). Targeted expression of human AML1-ETO fusion transcription factor in the haemocyte lineage cells by using the UAS/GAL4 system caused human leukaemic-like phenotypes such as hyper-proliferation of the circulating haemocytes resulting from the expansion of prohaemocytes in the LG (Sinenko et al., 2010). The successful establishment of the AML1-ETO leukemia model in *Drosophila* allowed a rapid tissue-specific genetic screening to identify suppressors for the hyperproliferation phenotype. The authors thus were able to show that ROS is a signaling factor promoting maintenance of normal as well as aberrant haemocyte precursors which suggested the importance of antioxidant enzymes and their regulators as targets for further study in the context of leukemia (Sinenko et al., 2010). In another independent genetic screening for modifiers in the AML1-ETO *Drosophila* model, Osman et al. identified calpainB as required for AML1-ETO-induced blood cell disorders in *Drosophila* by using an *in vivo* RNAi-based screen for suppressors of AML1-ETO. Remarkably, calpain was also found to interact with AML1-ETO in the human leukemic blood cell line Kasumi-1 (Osman et al., 2009). Therefore these studies have paved new avenues in our understanding of the pathogenesis of haematopoietic associated leukemia by developing and recapitulating the fundamental features of the disease in *Drosophila* and will contribute significantly to more precise and effective AML therapy.

#### *Drosophila* and human solid tumor

During the past two decades, the completion of the *Drosophila* genome and the development of advanced genome editing tools have given unprecedented stimulus and fast expansion on the use of *Drosophila* as a model for cancer. In particular, *Drosophila* has been instrumental for the discovery of three fundamental mechanisms involved in tumor progression and metastasis: (1) the role of Hippo signaling pathway in control of cell growth and survival together with Scrib/Dlg/Lgl signaling pathway in cell polarity to regulate organ sizes; (reviews in Enomoto and Igaki, 2011; Martin-Belmonte and Perez-Moreno, 2012; Harvey et al., 2013); (2) the *in situ* cell competition mechanism for morphogens during the formation of epithelium where slow growing cells are outcompeted and removed (review in Levayer and Moreno, 2013); and (3) apoptosis induced mitogenic signals in compensatory proliferation to replace surrounding damaged tissues (review in Fan and Bergmann, 2008). All these pathways underline the basic cellular communication, shaping and tissue organization which if disrupted can lead to tumorigenesis and facilitate tumor metastasis.

Despite a very short life span, cancer research to investigate directly the tumor progression and invasion in *Drosophila* can be dated back to nearly a century ago. It was observed that *Drosophila* can naturally develop hereditary tumors; carcinogens such as X-rays have been used to discover mutants with abnormal growth (Bridges and Brehme, 1944; Salomon and Jackson, 2008). The first fly strain carrying a mutation in a tumor suppressor gene called *lethal giant larvae* (*lgl*) was isolated 70 years ago. However only recently has Lgl been identified as a component of a signaling circuit in the regulation of apico-basal polarity in epithelial cells: Scrib/Dlg/Lgl (Humbert et al., 2008). Loss of function of the genes in this pathway can lead to the formation of neoplasma in the brain and imaginal disks in the developing larva. Based on the neoplastic phenotype observed in the scrib signaling mutants, Gateff was one of the pioneers who carried out more screenings to isolate recessive lethal mutations that could promote neoplastic overgrowth in the brain, imaginal disks or haematopoietic organ (Gateff, 1978). A tissue transplantation technique was developed from Gateff's study to assess the malignancy and invasive capacity of the neoplastic clones arising from various tissues. This technique involves the implantation of cancerous cell clones into the abdomen of wild type adult flies. Flies implanted with malignant cell clones usually die within 2 weeks and histological examination usually observes a massive invasion of cancerous cells into various tissues in the adult fly. This method resembles the tail veil intravenous injection of tumor cell lines into the mouse to model cancer in small rodents. This pioneering work fully established the potential for *Drosophila* to be used as a whole organism model to recapitulate key stages in cancer pathogenesis. The *in vivo* lac-Z reporter gene expression system can be utilized to quantify the proliferation rate and metastatic index of donor tumors in a tissue specific context in the normal wild type host (Beaucher et al., 2007). By using mutant clones of labeled cells such as GFP within a specific tissue, the invasive and metastatic behavior of cancer cells can be observed *in situ* by *in vivo* live imaging (Brumby and Richardson, 2003; Pagliarini and Xu, 2003). The recent development of MARCM system allows the establishment of large homozygous mutant cells clones within a normal or heterozygous tissue. A similar system is the FLP/FRT site directed recombination that can also generate genetic mosaics in the targeted organ or tissue. The mutant cells usually overexpress a UAS-tagged transgene fused with a reporter gene like GFP to allow imaging. These systems have been used by two independent groups to identify genes in cooperation with known oncogenes to induce tumor growth and invasion in the context of normal tissue (Brumby and Richardson, 2003; Pagliarini and Xu, 2003). By overexpressing an activated Ras (Ras^v12^) in the *Drosophila* eye imaginal disk and screening for the entire *Drosophila* genome, Pagliarini and Xu indentified Scrib as the promoting factor for the metastatic transformation of the otherwise benign tumor caused by overexpression of Ras oncogene alone (Pagliarini and Xu, 2003). Similar work by using the *Drosophila* eye as an *in vivo* “test tube” to investigate genetic interactions during tumor progression has proved to be extremely fruitful in dissecting the Scrib tumor suppressor signaling pathway and Hippo pathway and human oncogenes and tumor suppressors such as Ras and PTEN. Critical reviews of the most recent progress in the use of *Drosophila* in cancer modeling and therapeutic potentials can be found elsewhere (Miles et al., 2011; Stefanatos and Vidal, 2011; Gonzalez, 2013; Tipping and Perrimon, 2013).

#### Innate immunity in *Drosophila* tumor progression and tumor invasion model

Like other aspects of tumor progression that can be modeled and studied comparatively in *Drosophila*, the interplay of innate immunity mediated inflammation and tumor growth and invasion can be investigated accordingly. Of note is the establishment of *Drosophila* intestinal tumor model to study the molecular and cellular mechanisms linking inflammation and cancer pathogenesis (reviewed in Christofi and Apidianakis, 2013). The *Drosophila* cytokines Upd1-3 have been reported to activate the JAK/STAT signaling in promoting intestinal stem cell proliferation upon enteric infection or JNK-mediated stress response and thus the innate immunity plays essential roles in gut tissue homeostasis (Jiang et al., 2009). Moreover, overexpression of *Drosophila* Ras oncogene in association with bacterial infection can result in the formation of intestinal dysplasia (Apidianakis et al., 2009). More recently it is revealed that both the activation of Ras and bacterial induced IMD signaling can activate JNK pathway, which culminates in the up-regulation of matrix metalloproteinase 1 and thus cell invasion and migration (Bangi et al., 2012). Two studies have also emerged to explore the roles of haemocytes in the tumor progression and both relied on induced Ras^v12^Scrib^−/−^ model in the *Drosophila* eye disks, which has been used to offer complementary views of systemic innate immunity to tumor growth and invasion. Under the condition of established tumor, the *Drosophila* haemocyte could be recruited to the tumor site and tumor associated haemocytes are the major source of *Drosphila* TNFα, Eiger, to promote the growth and invasion of the tumor cells into other tissues (Cordero et al., 2010), while a TNF-dependent mechanism in *Drosophila* eliminates cells deficient for the polarity tumor suppressors Scrib or Dlg to maintain the tissue homeostasis and keep any malignant growth in check (Brumby and Richardson, 2003). In an earlier study by Tian Xu and co-workers, the same eye imaginal disk derived Ras^v12^Scrib^−/−^ tumor was found to induce a systemic proliferation of haemocytes via the JNK-JAK/STAT signaling cross-talk conserved also in response to tissue injury. The disrupted tumor basal membrane recruited circulating haemocytes in a manner reminiscence of wound healing (Pastor-Pareja et al., 2008). This study also modeled for the first time in *Drosophila* the anti-tumor effect of a systemic inflammation response induced by mechanical injury and provided critical insight into the cross-talk between the different signaling pathways in regulating the multiple step progression of tumor.

### Perspective

**Figure 3:**
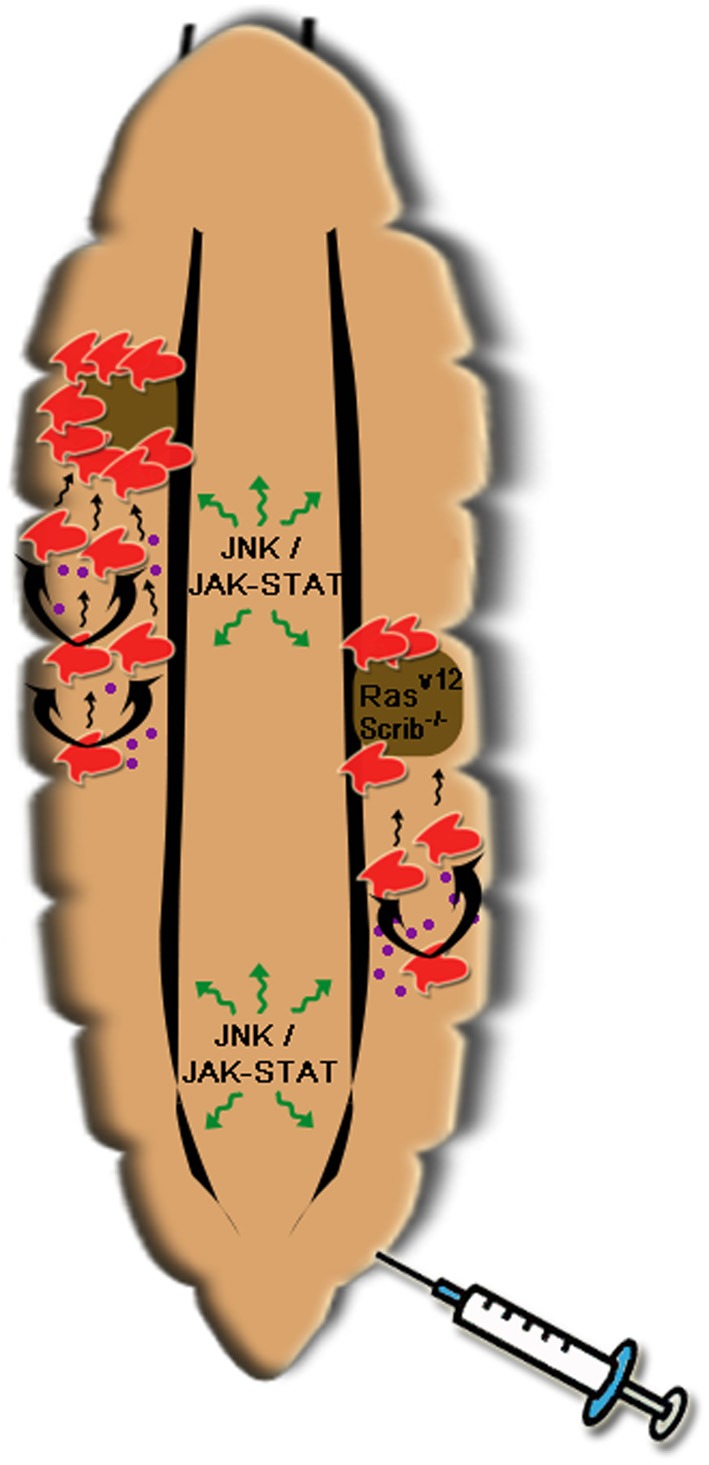
**A simple illustration of roles of haemocytes in the *Drosophila* tumor model**. Both tumor (Ras^v12^Scrib^−/−^) and wounding (as illustrated by a needle) can trigger proliferation of circulating haemocytes in the larva. This is mediated by cytokines (most probably Upd 1–3 shown in purple dots) induced by JNK signaling in response to wounding or the invasive tumor itself. In responses to the cytokines, JAK/STAT signaling in haemocytes or the fat body (not shown) can be activated and thus can further amplify the cytokine expression to promote haemocyte proliferation. Meanwhile disruption of basal membrane in the tumor attracts the adherent of haemocytes and limits the tumor growth perhaps by the synthesis of *Drosophila* TNFα, Eiger. This cartoon delineates that a step wise proliferation of haemocytes induced by wounding can be used to counter tumor growth. Modeling of these two processes (tumor growth and inflammation) in the *Drosophila* larva points out the potential beneficial effect that systemic inflammation can exert on tumorigenesis and will open more avenues for the basic research on innate immunity in particular macrophages' role in human tumor (see text for more detail).

Cancer has a complex biology. As a rationalization, Hanahan and Weinberg identified six major successive changes in human tumor development (Hanahan and Weinberg, 2011). The increasing awareness of this daunting complexity has turned more scientists to develop *Drosophila* as the complementary genetic tool to dissect each of the hallmark processes and to offer fundamental insights into the underlying genetic and cellular basis of the disease. In future, the *Drosophila* model can continue to be used as the reductionist system to investigate more of the cross-talks between these fundamental cancer biological processes. For example, the contrasting roles of immunity to control tumor growth or to be hijacked by the tumor can be modeled in the fly in a temporal and spatial manner. The unique advantage of *Drosophila* to be used as a whole organism has started to show promising potential in cancer drug screening and testing recently (Dar et al., 2012). This advantage can be fully explored by modeling simultaneously or sequentially physiological processes such as wound or infection induced innate immune/inflammatory response in the progression of cancer (Figure 3). The study of the role of haemocytes in response to wounding and invasive tumors will shed some light to the fundamentals of macrophages in immunity, inflammation and tumor microenvironment in human cancer.

### Conflict of interest statement

The authors declare that the research was conducted in the absence of any commercial or financial relationships that could be construed as a potential conflict of interest.

## Abbreviations

AMP: antimicrobial peptide
AML: acute myeloid leukemia
ALL: acute lymphoblastic leukemia
Bsk: Basket
Chn: Charlatan
CML: chronic myeloid leukemia
CNS: central nerve system
Col: Collier
Dif: Dorsal related immunity
Dom: Domeless
ECM: extracellular matrix proteins
EGF: epidermal growth factor
FOG: Friend of GATA
GADD45: growth arrest and DNA damage-inducible gene 45
Gcm: Glial-cells-missing
Hg: Hedgehog
Hml: Hemolectin
HSC: haematopoietic stem cells
JNK: Jun N-terminal kinase
LG: lymph gland
Lgl: lethal giant larvae
Lz: Lozenge
MARCM: mosaic analysis with a repressible cell marker system
PDGF: platelet-derived growth factor
PI3K: phosphatidyl-inositol 3-kinase
PPAE: Prophenoloxidase activating enzyme
PPO: Prophenoloxidase
PSC: posterior signaling center
ROS: reactive oxygen species
Ser: Serrate
Smo: Smoothened
Srp: Serpent
TF: Transglutaminase
Upd: Unpaired
Ush: U-shaped
VEGF: vascular endothelial growth factor
Wnt: Wingless.

## References

[B1] Abreu-Blanco M. T. Verboon J. M. Liu R. Watts J. J. Parkhurst S. M. (2012). *Drosophila* embryos close epithelial wounds using a combination of cellular protrusions and an actomyosin purse string. J. Cell. Sci. 125(Pt 24), 5984–5997 10.1242/jcs.109066 23038780 PMC3585516

[B2] Abreu-Blanco M. T. Verboon J. M. Parkhurst S. M. (2011). Cell wound repair in *Drosophila* occurs through three distinct phases of membrane and cytoskeletal remodeling. J. Cell. Biol. 193, 455–464 10.1083/jcb.201011018 21518790 PMC3087011

[B3] Agaisse H. Petersen U. M. Boutros M. Mathey-Prevot B. Perrimon N. (2003). Signaling role of hemocytes in *Drosophila* JAK/STAT-dependent response to septic injury. Dev. Cell. 5, 441–450 10.1016/S1534-5807(03)00244-2 12967563

[B4] Aggarwal B. B. Vijayalekshmi R. V, Sung B. (2009). Targeting inflammatory pathways for prevention and therapy of cancer: short-term friend, long-term foe. Clin. Cancer Res. 15, 425–430 10.1158/1078-0432 19147746

[B5] Apidianakis Y. Pitsouli C. Perrimon N. Rahme L. (2009). Synergy between bacterial infection and genetic predisposition in intestinal dysplasia. Proc. Natl. Acad. Sci. U.S.A. 106, 20883–20888 10.1073/pnas.0911797106 19934041 PMC2791635

[B6] Bangi E. Pitsouli C. Rahme L. G. Cagan R. Apidianakis Y. (2012). Immune response to bacteria induces dissemination of Ras-activated *Drosophila* hindgut cells. EMBO Rep. 13, 569–576 10.1038/embor.2012.44 22498775 PMC3367237

[B7] Bataillé L. Augé B. Ferjoux G. Haenlin M. Waltzer L. (2005). Resolving embryonic blood cell fate choice in *Drosophila*: interplay of GCM and RUNX factors. Development 132, 4635–4644 10.1242/dev.02034 16176949

[B8] Beaucher M. Goodliffe J. Hersperger E. Trunova S. Frydman H. Shearn A. (2007). *Drosophila* brain tumor metastases express both neuronal and glial cell type markers. Dev. Biol. 301, 287–297 10.1016/j.ydbio.2006.09.019 17055475 PMC1859848

[B9] Belacortu Y. Paricio N. (2011). *Drosophila* as a model of wound healing and tissue regeneration in vertebrates. Dev. Dyn. 240, 2379–2404 10.1002/dvdy.22753 21953647

[B10] Bergmann A. Steller H. (2010). Apoptosis, stem cells, and tissue regeneration. Sci. Signal. 3, re8 10.1126/scisignal.3145re8 20978240 PMC2991142

[B11] Bidla G. Dushay M. S. Theopold U. (2007). Crystal cell rupture after injury in *Drosophila* requires the JNK pathway, small GTPases and the TNF homolog Eiger. J. Cell. Sci. 120(Pt 7), 1209–1215 10.1242/jcs.03420 17356067

[B12] Braun A. Hoffmann J. A. Meister M. (1998). Analysis of the *Drosophila* host defense in domino mutant larvae, which are devoid of hemocytes. Proc. Natl. Acad. Sci. U.S.A. 95, 14337–14342 10.1073/pnas.95.24.14337 9826701 PMC24374

[B13] Brennan C. A. Delaney J. R. Schneider D. S. Anderson K. V. (2007). Psidin is required in *Drosophila* blood cells for both phagocytic degradation and immune activation of the fat body. Curr. Biol. 17, 67–72 10.1016/j.cub.2006.11.026 17208189

[B13a] Bridges C. B. Brehme K. S. (1944). The Mutants of Drosophila Melanogaster. Publs Carnegie Instn, 552: vii + 257pp

[B14a] Brückner K. Kockel L. Duchek P. Luque C. M. Rørth P. Perrimon N. (2004). The PDGF/VEGF receptor controls blood cell survival in *Drosophila*. Dev. Cell 1, 73–84 10.1016/j.devcel.2004.06.007 15239955

[B14] Brumby A. M. Richardson H. E. (2003). scribble mutants cooperate with oncogenic Ras or Notch to cause neoplastic overgrowth in *Drosophila*. EMBO J. 22, 5769–5779 10.1093/emboj/cdg548 14592975 PMC275405

[B15] Candido J. Hagemann T. (2013). Cancer-related inflammation. J. Clin. Immunol. 33, S79–S84 10.1007/s10875-012-9847-0 23225204

[B16] Castillejo-López C. Häcker U. (2005). The serine protease Sp7 is expressed in blood cells and regulates the melanization reaction in *Drosophila*. Biochem. Biophys. Res. Commun 338, 1075–1082 10.1016/j.bbrc.2005.10.042 16256951

[B17] Cerenius L. Söderhäll K. (2004). The prophenoloxidase-activating system in invertebrates. Immunol. Rev. 198, 116–126 10.1111/j.0105-2896.2004.00116.x 15199959

[B18] Charroux B. Royet J. (2009). Elimination of plasmatocytes by targeted apoptosis reveals their role in multiple aspects of the *Drosophila* immune response. Proc. Natl. Acad. Sci. U.S.A. 106, 9797–9802 10.1073/pnas.0903971106 19482944 PMC2700997

[B19] Cho N. K. Keyes L. Johnson E. Heller J. Ryner L. Karim F. (2002). Developmental control of blood cell migration by the *Drosophila* VEGF pathway. Cell 108, 865–876 10.1016/S0092-8674(02)00676-1 11955438

[B20] Christofi T. Apidianakis Y. (2013). Ras-oncogenic *Drosophila* hindgut but not midgut cells use an inflammation-like program to disseminate to distant sites. Gut. Microbes 4, 54–59 10.4161/gmic.22429 23060054 PMC3555887

[B21] Corbo J. C. Levine M. (1996). Characterization of an immunodeficiency mutant in *Drosophila*. Mech. Dev. 55, 211–220 10.1016/0925-4773(96)00506-0 8861100

[B22] Cordero J. B. Macagno J. P. Stefanatos R. K. Strathdee K. E. Cagan R. L. Vidal M. (2010). Oncogenic Ras diverts a host TNF tumor suppressor activity into tumor promoter. Dev. Cell. 18, 999–1011 10.1016/j.devcel.2010.05.014 20627081 PMC3175220

[B23] Coussens L. M. Werb Z. (2002). Inflammation and cancer. Nature 420, 860–867 10.1038/nature01322 12490959 PMC2803035

[B24] Cronin S. J. F. Nehme N. T. Limmer S. Liegeois S. Pospisilik J. A. Schramek D. (2009). Genome-wide RNAi screen identifies genes involved in intestinal pathogenic bacterial infection. Science 325, 340–343 10.1126/science.1173164 19520911 PMC2975362

[B25] Crozatier M. Meister M. (2007). *Drosophila* haematopoiesis. Cell. Microbiol. 9, 1117–1126 10.1111/j.1462-5822.2007.00930.x 17394559

[B26] Crozatier M. Ubeda J.-M. Vincent A. Meister M. (2004). Cellular immune response to parasitization in *Drosophila* requires the EBF ortholog collier. PLoS Biol. 2:E196 10.1371/journal.pbio.0020196 15314643 PMC509289

[B27] Crozatier M. Vincent A. (2011). *Drosophila*: a model for studying genetic and molecular aspects of haematopoiesis and associated leukaemias. Dis. Model Mech. 4, 439–445 10.1242/dmm.007351 21669932 PMC3124048

[B28] Cuttell L. Vaughan A. Silva E. Escaron C. J. Lavine M. Van Goethem E. (2008). Undertaker, a *Drosophila* Junctophilin, links Draper-mediated phagocytosis and calcium homeostasis. Cell 135, 524–534 10.1016/j.cell.2008.08.033 18984163

[B29] Dar A. C. Das T. K. Shokat K. M. Cagan R. L. (2012). Chemical genetic discovery of targets and anti-targets for cancer polypharmacology. Nature 486, 80–84 10.1038/nature11127 22678283 PMC3703503

[B30] De Gregorio E. Han S.-J. Lee W.-J. Baek M.-J. Osaki T. Kawabata S. (2002). An immune-responsive Serpin regulates the melanization cascade in *Drosophila*. Dev. Cell. 3, 581–592 10.1016/S1534-5807(02)00267-8 12408809

[B31] De Palma M. Lewis C. E. (2013). Macrophage regulation of tumor responses to anticancer therapies. Cancer Cell 23, 277–286 10.1016/j.ccr.2013.02.013 23518347

[B32] Defaye A. Evans I. Crozatier M. Wood W. Lemaitre B. Leulier F. (2009). Genetic ablation of *Drosophila* phagocytes reveals their contribution to both development and resistance to bacterial infection. J. Innate Immun. 1, 322–334 10.1159/000210264 20375589

[B33] Dierks C. Grbic J. Zirlik K. Beigi R. Englund N. P. Guo G. R. (2007). Essential role of stromally induced hedgehog signaling in B-cell malignancies. Nat. Med. 13, 944–951 10.1038/nm1614 17632527

[B34] Dragojlovic-Munther M. Martinez-Agosto J. A. (2012). Multifaceted roles of PTEN and TSC orchestrate growth and differentiation of *Drosophila* blood progenitors. Development 139, 3752–3763 10.1242/dev.074203 22951642 PMC3445307

[B35] Dunn G. P. Old L. J. Schreiber R. D. (2004). The immunobiology of cancer immunosurveillance and immunoediting. Immunity 21, 137–148 10.1016/j.immuni.2004.07.017 15308095

[B36] Ekas L. A Cardozo T. J. Flaherty M. S. McMillan E. A Gonsalves F. C. Bach E. A. (2010). Characterization of a dominant-active STAT that promotes tumorigenesis in *Drosophila*. Dev. Biol. 344, 621–636 10.1016/j.ydbio.2010.05.497 20501334 PMC2914209

[B37] Eleftherianos I. Revenis C. (2011). Role and importance of phenoloxidase in insect hemostasis. J. Innate Immun. 3, 28–33 10.1159/000321931 21051882

[B38] Enomoto M. Igaki T. (2011). Deciphering tumor-suppressor signaling in flies: genetic link between Scribble/Dlg/Lgl and the Hippo pathways. J. Genet. Genomics 38, 461–470 10.1016/j.jgg.2011.09.005 22035867

[B39] Evans C. J. Hartenstein V. Banerjee U. (2003). Thicker than blood: conserved mechanisms in *Drosophila* and vertebrate hematopoiesis. Dev. Cell. 5, 673–690 10.1016/S1534-5807(03)00335-6 14602069

[B40] Fan Y. Bergmann A. (2008). Apoptosis-induced compensatory proliferation. The Cell is dead. Long live the Cell! Trends Cell. Biol. 18, 467–473 10.1016/j.tcb.2008.08.001 18774295 PMC2705980

[B41] Fossett N. Tevosian S. G. Gajewski K. Zhang Q. Orkin S. H. Schulz R. A. (2001). The Friend of GATA proteins U-shaped, FOG-1, and FOG-2 function as negative regulators of blood, heart, and eye development in *Drosophila*. Proc. Natl. Acad. Sci. U.S.A. 98, 7342–7347 10.1073/pnas.131215798 11404479 PMC34670

[B42] Franc N. C. Dimarcq J. L. Lagueux M. Hoffmann J. Ezekowitz R. A. (1996). Croquemort, a novel *Drosophila* hemocyte/macrophage receptor that recognizes apoptotic cells. Immunity 4, 431–443 10.1016/S1074-7613(00)80410-0 8630729

[B43] Galko M. J. Krasnow M. A. (2004). Cellular and genetic analysis of wound healing in *Drosophila* larvae. PLoS Biol. 2:E239 10.1371/journal.pbio.0020239 15269788 PMC479041

[B44] Gandillet, A, Park S. Lassailly F. Griessinger E. Vargaftig J. Filby A. (2011). Heterogeneous sensitivity of human acute myeloid leukemia to β-catenin down-modulation. Leukemia 25, 770–780 10.1038/leu.2011.17 21339756 PMC4289854

[B45] Gateff E. (1978). Malignant neoplasms of genetic origin in *Drosophila melanogaster*. Science 200, 1448–1459 10.1126/science.96525 96525

[B46] Geissler K. Zach O. (2012). Pathways involved in *Drosophila* and human cancer development: the Notch, Hedgehog, Wingless, Runt, and Trithorax pathway. Ann. Hematol. 91, 645–669 10.1007/s00277-012-1435-0 22418742

[B47] Glittenberg M. T. Kounatidis I. Christensen D. Kostov M. Kimber S. Roberts I. (2011). Pathogen and host factors are needed to provoke a systemic host response to gastrointestinal infection of *Drosophila* larvae by *Candida albicans*. Dis. Model Mech. 4, 515–525 10.1242/dmm.006627 21540243 PMC3124059

[B48] Gonzalez C. (2013). *Drosophila melanogaster*: a model and a tool to investigate malignancy and identify new therapeutics. Nat. Rev. Cancer 13, 172–183 10.1038/nrc3461 23388617

[B49] Goto A. Kadowaki T. Kitagawa Y. (2003). Drosophila hemolectin gene is expressed in embryonic and larval hemocytes and its knock down causes bleeding defects. Dev. Biol. 264, 582–591 10.1016/j.ydbio.2003.06.001 14651939

[B50] Goto A. Kumagai T. Kumagai C. Hirose J. Narita H. Mori H. (2001). A *Drosophila* haemocyte-specific protein, hemolectin, similar to human von Willebrand factor. Biochem. J. 359, 99–108 10.1042/0264-6021:3590099 11563973 PMC1222125

[B51] Grivennikov S. I. Greten F. R. Karin M. (2010). Immunity, inflammation, and cancer. Cell 140, 883–899 10.1016/j.cell.2010.01.025 20303878 PMC2866629

[B52] Hanahan D. Weinberg R. A. (2011). Hallmarks of cancer: the next generation. Cell 144, 646–674 10.1016/j.cell.2011.02.013 21376230

[B53] Harris H. (2005). A long view of fashions in cancer research. Bioessays 27, 833–838 10.1002/bies.20263 16015588

[B54] Harrison D. A. Binari R. Nahreini T. S. Gilman M. Perrimon N. (1995). Activation of a *Drosophila* Janus kinase (JAK) causes hematopoietic neoplasia and developmental defects. EMBO J. 14, 2857–2865 7796812 10.1002/j.1460-2075.1995.tb07285.x PMC398404

[B55] Hartenstein V. (2006). Blood cells and blood cell development in the animal kingdom. Annu. Rev. Cell. Dev. Biol. 22, 677–712 10.1146/annurev.cellbio.22.010605.093317 16824014

[B56] Harvey K. F. Zhang X. Thomas D. M. (2013). The Hippo pathway and human cancer. Nat. Rev. Cancer 13, 246–257 10.1038/nrc3458 23467301

[B57] Hashimoto Y. Tabuchi Y. Sakurai K. Kutsuna M. Kurokawa K. Awasaki T. (2009). Identification of lipoteichoic acid as a ligand for draper in the phagocytosis of Staphylococcus aureus by *Drosophila* hemocytes. J. Immunol. 183, 7451–7460 10.4049/jimmunol.0901032 19890048

[B58] Hatlen M. A. Wang L. Nimer S. D. (2012). AML1-ETO driven acute leukemia: insights into pathogenesis and potential therapeutic approaches. Front. Med. 6:3 10.1007/s11684-012-0206-6 22875638

[B59] Holz A. Bossinger B. Strasser T. Janning W. Klapper R. (2003). The two origins of hemocytes in *Drosophila*. Development 130, 4955–4962 10.1242/dev.00702 12930778

[B60] Howell L. Sampson C. J. Xavier M. J. Bolukbasi E. Heck M. M. S. Williams M. J. (2012). A directed miniscreen for genes involved in the *Drosophila* anti-parasitoid immune response. Immunogenetics 64, 155–161 10.1007/s00251-011-0571-3 21947570

[B61] Hsu T. (2012). Complex cellular functions of the von Hippel-Lindau tumor suppressor gene: insights from model organisms. Oncogene 31, 2247–2257 10.1038/onc.2011.442 21996733 PMC3343179

[B62] Huelsmann S. Hepper C. Marchese D. Knöll C. Reuter R. (2006). The PDZ-GEF dizzy regulates cell shape of migrating macrophages via Rap1 and integrins in the *Drosophila* embryo. Development 133, 2915–2924 10.1242/dev.02449 16818452

[B63] Humbert P. O. Grzeschik N. A. Brumby A. M. Galea R. Elsum I. Richardson H. E. (2008). Control of tumourigenesis by the Scribble/Dlg/Lgl polarity module. Oncogene 27, 6888–6907 10.1038/onc.2008.341 19029932

[B64] Irving P. Ubeda J. M. Doucet D. Troxler L. Lagueux M. Zachary D. (2005). New insights into *Drosophila* larval haemocyte functions through genome-wide analysis. Cell. Microbiol. 7, 335–350 10.1111/j.1462-5822.2004.00462.x 15679837

[B65] Jiang H. Patel P. H. Kohlmaier A. Grenley M. O. McEwen D. G. Edgar B. A. (2009). Cytokine/Jak/Stat signaling mediates regeneration and homeostasis in the *Drosophila* midgut. Cell 137, 1343–1355 10.1016/j.cell.2009.05.014 19563763 PMC2753793

[B66] Jiravanichpaisal P. Lee B. L. Söderhäll K. (2006). Cell-mediated immunity in arthropods: hematopoiesis, coagulation, melanization and opsonization. Immunobiology 211, 213–236 10.1016/j.imbio.2005.10.015 16697916

[B67] Johansson K. C. Metzendorf C. Söderhäll K. (2005). Microarray analysis of immune challenged *Drosophila* hemocytes. Exp. Cell. Res. 305, 145–155 10.1016/j.yexcr.2004.12.018 15777795

[B68] Jung S. H. Evans C. J. Uemura C. Banerjee U. (2005). The *Drosophila* lymph gland as a developmental model of hematopoiesis. Development 132, 2521–2533 10.1242/dev.01837 15857916

[B69] Karlsson C. Korayem A. M. Scherfer C. Loseva O. Dushay M. S. Theopold U. (2004). Proteomic analysis of the *Drosophila* larval hemolymph clot. J. Biol. Chem. 279, 52033–52041 10.1074/jbc.M408220200 15466469

[B70] Kiehart D. P. Galbraith C. G. Edwards K. A. Rickoll W. L. Montague R. A. (2000). Multiple forces contribute to cell sheet morphogenesis for dorsal closure in *Drosophila*. J. Cell. Biol. 149, 471–490 10.1083/jcb.149.2.471 10769037 PMC2175161

[B71] Kocks C. Cho J. H. Nehme N. Ulvila J. Pearson A. M. Meister M. (2005). Eater, a transmembrane protein mediating phagocytosis of bacterial pathogens in *Drosophila*. Cell 123, 335–346 10.1016/j.cell.2005.08.034 16239149

[B72] Kounatidis I. Ligoxygakis P. (2012). *Drosophila* as a model system to unravel the layers of innate immunity to infection. Open Biol. 2, 120075 10.1098/rsob.120075 22724070 PMC3376734

[B73] Krzemien J. Crozatier M. Vincent A. (2010). Ontogeny of the *Drosophila* larval hematopoietic organ, hemocyte homeostasis and the dedicated cellular immune response to parasitism. Int. J. Dev. Biol. 54, 1117–1125 10.1387/ijdb.093053jk 20711989

[B74] Krzemień J. Dubois L. Makki R. Meister M. Vincent A. Crozatier M. (2007). Control of blood cell homeostasis in *Drosophila* larvae by the posterior signalling centre. Nature 446, 325–328 10.1038/nature05650 17361184

[B75] Kurant E. Axelrod S. Leaman D. Gaul U. (2008). Six-microns-under acts upstream of Draper in the glial phagocytosis of apoptotic neurons. Cell 133, 498–509 10.1016/j.cell.2008.02.052 18455990 PMC2730188

[B76] Kurucz E. Márkus R. Zsámboki J. Folkl-Medzihradszky K. Darula Z. Vilmos P. (2007). Nimrod, a putative phagocytosis receptor with EGF repeats in *Drosophila* plasmatocytes. Curr. Biol. 17, 649–654 10.1016/j.cub.2007.02.041 17363253

[B77] Kurucz E. Zettervall C. Sinka R. Vilmos P. Pivarcsi A. Ekengren S. (2003). Hemese, a hemocyte-specific transmembrane protein, affects the cellular immune response in Drosophila. Proc. Natl. Acad. Sci. U.S.A. 100, 2622–2627 10.1073/pnas.0436940100 12598653 PMC151390

[B78] Lacronique V. Boureux A. Valle V. D. Poirel H. Quang C. T. Mauchauffé M. (1997). A TEL-JAK2 fusion protein with constitutive kinase activity in human leukemia. Science 278, 1309–1312 10.1126/science.278.5341.1309 9360930

[B79] Lanot R. Zachary D. Holder F. Meister M. (2001). Postembryonic hematopoiesis in *Drosophila*. Dev. Biol. 230, 243–257 10.1006/dbio.2000.0123 11161576

[B80] Lebestky T. Chang T. Hartenstein V. Banerjee U. (2000). Specification of *Drosophila* hematopoietic lineage by conserved transcription factors. Science 288, 146–149 10.1126/science.288.5463.146 10753120

[B81] Lebestky T. Jung S. H. Banerjee U. (2003). A Serrate-expressing signaling center controls *Drosophila* hematopoiesis. Genes Dev. 17, 348–353 10.1101/gad.1052803 12569125 PMC195988

[B82] Leclerc V. Pelte N. El Chamy L. Martinelli C. Ligoxygakis P. Hoffmann J. A. (2006). Prophenoloxidase activation is not required for survival to microbial infections in *Drosophila*. EMBO Rep. 7, 231–235 10.1038/sj.embor.7400592 16322759 PMC1369246

[B83] Lee H. J. Daver N. Kantarjian H. M. Verstovsek S. Ravandi F. (2013a). The role of JAK pathway dysregulation in the pathogenesis and treatment of acute myeloid leukemia. Clin. Cancer Res. 19, 327–335 10.1158/1078-0432.CCR-12-2087 23209034

[B84] Lee H. W. Choi H. J. Ha S. J. Lee K. T. Kwon Y. G. (2013b). Recruitment of monocytes/macrophages in different tumor microenvironments. Biochim. Biophys. Acta 1835, 170–179 10.1016/j.bbcan.2012.12.007 23287570

[B85] Lee M. J. Kalamarz M. E. Paddibhatla I. Small C. Rajwani R. Govind S. (2009). Virulence factors and strategies of Leptopilina spp.: selective responses in *Drosophila* hosts. Adv. Parasitol. 70, 123–145 10.1016/S0065-308X(09)70005-3 19773069 PMC3363966

[B86] Lemaitre B. Hoffmann J. (2007). The host defense of *Drosophila melanogaster*. Annu. Rev. Immunol. 25, 697–743 10.1146/annurev.immunol.25.022106.141615 17201680

[B87] Lensch M. W. (2012). An evolving model of hematopoietic stem cell functional identity. Stem Cell Rev. 8, 551–560 10.1007/s12015-012-9347-x 22278132

[B88] Lesch C. Jo J. Wu Y. Fish G. S. Galko M. J. (2010). A targeted UAS-RNAi screen in *Drosophila* larvae identifies wound closure genes regulating distinct cellular processes. Genetics 186, 943–957 10.1534/genetics.110.121822 20813879 PMC2975283

[B89] Levayer R. Moreno E. (2013). Mechanisms of cell competition: themes and variations. J. Cell. Biol. 200, 689–698 10.1083/jcb.201301051 23509066 PMC3601360

[B90] Ligoxygakis P. Pelte N. Ji C. Leclerc V. Duvic B. Belvin M. (2002). A serpin mutant links Toll activation to melanization in the host defence of *Drosophila*. EMBO J. 21, 6330–6337 10.1093/emboj/cdf661 12456640 PMC136964

[B91] Lin J. Hackam D. J. (2011). Worms, flies and four-legged friends: the applicability of biological models to the understanding of intestinal inflammatory diseases. Dis. Model. Mech. 4, 447–456 10.1242/dmm.007252 21669933 PMC3124049

[B92] Lindgren M. Riazi R. Lesch C. Wilhelmsson C. Theopold U. Dushay M. S. (2008). Fondue and transglutaminase in the *Drosophila* larval clot. J. Insect Physiol. 54, 586–592 10.1016/j.jinsphys.2007.12.008 18222466

[B93] Manaka J. Kuraishi T. Shiratsuchi A. Nakai Y. Higashida H. Henson P. (2004). Draper-mediated and phosphatidylserine-independent phagocytosis of apoptotic cells by *Drosophila* hemocytes/macrophages. J. Biol. Chem. 279, 48466–48476 10.1074/jbc.M408597200 15342648

[B94] Mandal L. Banerjee U. Hartenstein V. (2004). Evidence for a fruit fly hemangioblast and similarities between lymph-gland hematopoiesis in fruit fly and mammal aorta-gonadal-mesonephros mesoderm. Nat. Genet. 36, 1019–1023 10.1038/ng1404 15286786

[B95] Mandal L. Martinez-Agosto J. A. Evans C. J. Hartenstein V. Banerjee U. (2007). A Hedgehog- and Antennapedia-dependent niche maintains *Drosophila* haematopoietic precursors. Nature 446, 320–324 10.1038/nature05585 17361183 PMC2807630

[B96] Mantovani A. Allavena P. Sica A. Balkwill F. (2008). Cancer-related inflammation. Nature 454, 436–444 10.1038/nature07205 18650914

[B97] Mantovani A. Biswas S. K. Galdiero M. R. Sica A. Locati M. (2013). Macrophage plasticity and polarization in tissue repair and remodelling. J. Pathol. 229, 176–185 10.1002/path.4133 23096265

[B98] Martelli A. M. Evangelisti C. Chappell W. Abrams S. L. Bäsecke J. Stivala F. (2011). Targeting the translational apparatus to improve leukemia therapy: roles of the PI3K/PTEN/Akt/mTOR pathway. Leukemia 25, 1064–1079 10.1038/leu.2011.46 21436840

[B99] Martin P. Lewis J (1992). Actin cables and epidermal movement in embryonic wound healing. Nature 360, 179–183 10.1038/360179a0 1436096

[B100] Martin-Belmonte F. Perez-Moreno M. (2012). Epithelial cell polarity, stem cells and cancer. Nat. Rev. Cancer 12, 23–38 10.1038/nrc3169 22169974

[B101] Miles W. O. Dyson N. J. Walker J. A. (2011). Modeling tumor invasion and metastasis in *Drosophila*. Dis. Model Mech. 4, 753–761 10.1242/dmm.006908 21979943 PMC3209645

[B102] Moreira C. G. A Jacinto A. Prag S. (2013). *Drosophila* integrin adhesion complexes are essential for hemocyte migration *in vivo*. Biol. Open 2, 795–801 10.1242/bio.20134564 23951405 PMC3744071

[B103] Moreira S. Stramer B. Evans I. Wood W. Martin P. (2010). Prioritization of competing damage and developmental signals by migrating macrophages in the *Drosophila* embryo. Curr. Biol. 20, 464–470 10.1016/j.cub.2010.01.047 20188558

[B104] Mukherjee T. Kim W. S. Mandal L. Banerjee U. (2011). Interaction between Notch and Hif-alpha in development and survival of *Drosophila* blood cells. Science 332, 1210–1213 10.1126/science.1199643 21636775 PMC4412745

[B105] Müller P. Kuttenkeuler D. Gesellchen V. Zeidler M. P. Boutros M. (2005). Identification of JAK/STAT signalling components by genome-wide RNA interference. Nature 436, 871–875 10.1038/nature03869 16094372

[B106] Nagao R. Ashihara E. Kimura S. Strovel J. W. Yao H. Takeuchi M. (2011). Growth inhibition of imatinib-resistant CML cells with the T315I mutation and hypoxia-adaptation by AV65–a novel Wnt/β-catenin signaling inhibitor. Cancer Lett. 312, 91–100 10.1016/j.canlet.2011.08.002 21906872

[B107] Nam H. J. Jang I. H. You H. Lee K. A. Lee W. J. (2012). Genetic evidence of a redox-dependent systemic wound response via Hayan protease-phenoloxidase system in *Drosophila*. EMBO J. 31, 1253–1265 10.1038/emboj.2011.476 22227521 PMC3297987

[B108] Nappi A. J. Vass E. Frey F. Carton Y. (1995). Superoxide anion generation in *Drosophila* during melanotic encapsulation of parasites. Eur. J. Cell. Biol. 68, 450–456 8690025

[B109] Novak M. L. Koh T. J. (2013). Macrophage phenotypes during tissue repair. J. Leukoc. Biol. 93, 875–881 10.1189/jlb.1012512 23505314 PMC3656331

[B110] Of B. Healing W. (1999). Cutaneous wound healing. N. Engl. J. Med. 738–746 10.1056/NEJM199909023411006 10471461

[B111] Oh I. H. Humphries R. K. (2012). Concise review: multidimensional regulation of the hematopoietic stem cell state. Stem Cells 30, 82–88 10.1002/stem.776 22083966

[B112] Osman D. Gobert V. Ponthan F. Heidenreich O. Haenlin M. Waltzer L. (2009). A *Drosophila* model identifies calpains as modulators of the human leukemogenic fusion protein AML1-ETO. Proc. Natl. Acad. Sci. U.S.A. 106, 12043–12048 10.1073/pnas.0902449106 19581587 PMC2715513

[B113] Owusu-Ansah E. Banerjee U. (2009). Reactive oxygen species prime *Drosophila* haematopoietic progenitors for differentiation. Nature 461, 537–541 10.1038/nature08313 19727075 PMC4380287

[B114] Pagliarini R. A. Xu T. (2003). A genetic screen in *Drosophila* for metastatic behavior. Science 302, 1227–1231 10.1126/science.1088474 14551319

[B115] Paladi M. Tepass U. (2004). Function of Rho GTPases in embryonic blood cell migration in *Drosophila*. J. Cell. Sci. 117(Pt 26), 6313–6326 10.1242/jcs.01552 15561773

[B116] Panayidou S. Apidianakis Y. (2013). Regenerative inflammation: lessons from *Drosophila* intestinal epithelium in health and disease Pathogens 2, 209–231 10.3390/pathogen2020209 PMC4235722 25437036

[B117] Pancewicz J. Nicot C. (2011). Current views on the role of Notch signaling and the pathogenesis of human leukemia. BMC Cancer 11:502 10.1186/1471-2407-11-502 22128846 PMC3262490

[B118] Pastor-Pareja J. C. Wu M. Xu T. (2008). An innate immune response of blood cells to tumors and tissue damage in *Drosophila*. Dis. Model Mech. 1, 144–154 10.1242/dmm.000950 19048077 PMC2562178

[B119] Peeples E. E. Geisler A. Whitcraft C. J. Oliver C. (1969). Comparative studies of phenol oxidase activity during pupal development of three lozenge mutants (lzs, lz, lzk) of *Drosophila melanogaster*. Genetics 62, 161–170 4984328 10.1093/genetics/62.1.161 PMC1212259

[B120] Qiu P. Pan P. C. Govind S. (1998). A role for the *Drosophila* Toll/Cactus pathway in larval hematopoiesis. Development 125, 1909–1920 9550723 10.1242/dev.125.10.1909

[B121] Radtke F. Wilson A. MacDonald H. R. (2005). Notch signaling in hematopoiesis and lymphopoiesis: lessons from *Drosophila*. BioEssays 27, 1117–1128 10.1002/bies.20315 16237675

[B122] Rämet M. Lanot R. Zachary D. Manfruelli P. (2002). JNK signaling pathway is required for efficient wound healing in *Drosophila*. Dev. Biol. 241, 145–156 10.1006/dbio.2001.0502 11784101

[B123] Razzell W. Evans I. R. Martin P. Wood W. (2013). Calcium flashes orchestrate the wound inflammatory response through DUOX activation and hydrogen peroxide release. Curr. Biol. 23, 424–429 10.1016/j.cub.2013.01.058 23394834 PMC3629559

[B124] Rezaie A. Parker R. D. Abdollahi M. (2007). Oxidative stress and pathogenesis of inflammatory bowel disease: an epiphenomenon or the cause? Dig. Dis. Sci. 52, 2015–2021 10.1007/s10620-006-9622-2 17404859

[B125] Ricklin D. Lambris J. D. (2013). Complement in immune and inflammatory disorders: pathophysiological mechanisms. J. Immunol. 190, 3831–3838 10.4049/jimmunol.1203487 23564577 PMC3623009

[B126] Ríos-Barrera L. D. Riesgo-Escovar J. R. (2013). Regulating cell morphogenesis: the *Drosophila* Jun N-terminal kinase pathway. Genesis 51, 147–162 10.1002/dvg.22354 23109363

[B127] Rizki R. M. Rizki T. M. (1980). Hemocyte responses to implanted tissues in *Drosophila melanogaster* larvae. Rouxs Arch. Dev. Biol. 213, 207–213 10.1007/BF00868679 28305176

[B128] Rizki T. M. Rizki R. M. Grell E. H. (1980). A mutant affecting the crystal cells in *Drosophila melanogaster*. Rouxs Arch. Dev. Biol. 181, 91–99 10.1007/BF00848799 28304971

[B129] Royet J. (2011). Epithelial homeostasis and the underlying molecular mechanisms in the gut of the insect model *Drosophila melanogaster*. Cell. Mol. Life Sci. 68, 3651–3660 10.1007/s00018-011-0828-x 21964927 PMC11115164

[B130] Salomon R. N. Jackson F. R. (2008). Tumors of testis and midgut in aging flies. Fly 2, 265–268 19077545 10.4161/fly.7396

[B131] Sampson C. J. Valanne S. Fauvarque M.-O. Hultmark D. Rämet M. Williams M. J. (2012). The RhoGEF Zizimin-related acts in the *Drosophila* cellular immune response via the Rho GTPases Rac2 and Cdc42. Dev. Comp. Immunol. 38, 160–168 10.1016/j.dci.2012.05.004 22634526

[B132] Sampson C. J. Williams M. J. (2012). Real-time analysis of *Drosophila* post-embryonic haemocyte behaviour. PLoS ONE 7:e28783 10.1371/journal.pone.0028783 22242151 PMC3252279

[B133] Scherfer C. Karlsson C. Loseva O. Bidla G. Goto A. Havemann J. (2004). Isolation and characterization of hemolymph clotting factors in *Drosophila melanogaster* by a pullout method. Curr. Biol. 14, 625–629 10.1016/j.cub.2004.03.030 15062105

[B134] Scherfer C. Qazi M. R. Takahashi K. Ueda R. Dushay M. S. Theopold U. (2006). The Toll immune-regulated *Drosophila* protein Fondue is involved in hemolymph clotting and puparium formation. Dev. Biol. 295, 156–163 10.1016/j.ydbio.2006.03.019 16690050

[B135] Schmidt R. L. Trejo T. R. Plummer T. B. Platt J. L. Tang A. H. (2008). Infection-induced proteolysis of PGRP-LC controls the IMD activation and melanization cascades in *Drosophila*. FASEB J. 22, 918–929 10.1096/fj.06-7907com 18308747

[B136] Schmucker D. Chen B. (2009). Dscam and DSCAM: complex genes in simple animals, complex animals yet simple genes. Genes Dev. 23, 147–156 10.1101/gad.1752909 19171779

[B137] Seke Etet P. F. Vecchio L. Bogne Kamga P. Nchiwan Nukenine E. Krampera M. Nwabo Kamdje A. H. (2013). Normal hematopoiesis and hematologic malignancies: role of canonical Wnt signaling pathway and stromal microenvironment. Biochim. Biophys. Acta. 1835, 1–10 10.1016/j.bbcan.2012.08.002 22982245

[B138] Sethi G. Shanmugam M. K. Ramachandran L. Kumar A. P. Tergaonkar V. (2012). Multifaceted link between cancer and inflammation. Biosci. Rep. 32, 1–15 10.1042/BSR20100136 21981137

[B139] Shia A. K. H. Glittenberg M. Thompson G. Weber A. N. Reichhart J. M. Ligoxygakis P. (2009). Toll-dependent antimicrobial responses in *Drosophila* larval fat body require Spätzle secreted by haemocytes. J. Cell. Sci. 122(Pt 24), 4505–4515 10.1242/jcs.049155 19934223 PMC2787462

[B140] Siekhaus D. Haesemeyer M. Moffitt O. Lehmann R. (2010). RhoL controls invasion and Rap1 localization during immune cell transmigration in *Drosophila*. Nat. Cell. Biol. 12, 605–610 10.1038/ncb2063 20495554 PMC3006444

[B141] Sinenko S. A Hung T. Moroz T. Tran Q. M. Sidhu S. Cheney M. D. (2010). Genetic manipulation of AML1-ETO-induced expansion of hematopoietic precursors in a *Drosophila* model. Blood 116, 4612–4620 10.1182/blood-2010-03-276998 20688956 PMC2996118

[B142] Sinenko S. A. Mandal L. Martinez-Agosto J. A. Banerjee U. (2009). Dual role of wingless signaling in stem-like hematopoietic precursor maintenance in *Drosophila*. Dev. Cell. 16, 756–763 10.1016/j.devcel.2009.03.003 19460351 PMC2718753

[B142a] Singer A. J. Clark R. A. (1999). Cutaneous wound healing. N. Engl. J. Med. 341, 738–746 10.1197/j.aem.2006.03.189 10471461

[B143] Sorrentino R. P. Carton Y. Govind S. (2002). Cellular immune response to parasite infection in the *Drosophila* lymph gland is developmentally regulated. Dev. Biol. 243, 65–80 10.1006/dbio.2001.0542 11846478

[B144] Sorrentino R. P. Melk J. P. Govind S. (2004). Genetic analysis of contributions of dorsal group and JAK-Stat92E pathway genes to larval hemocyte concentration and the egg encapsulation response in *Drosophila*. Genetics 166, 1343–1356 10.1534/genetics.166.3.1343 15082553 PMC1470785

[B145] Stefanatos R. K. A, Vidal M. (2011). Tumor invasion and metastasis in *Drosophila*: a bold past, a bright future. J. Genet. Genomics 38, 431–438 10.1016/j.jgg.2011.09.004 22035864

[B146] Stofanko M. Kwon S. Y. Badenhorst P. (2010). Lineage tracing of lamellocytes demonstrates *Drosophila* macrophage plasticity. PLoS ONE 5:e14051 10.1371/journal.pone.0014051 21124962 PMC2988793

[B147] Stramer B. Winfield M. Shaw T. Millard T. H. Woolner S. Martin P. (2008). Gene induction following wounding of wild-type versus macrophage-deficient *Drosophila* embryos. EMBO Rep. 9, 465–471 10.1038/embor.2008.34 18344972 PMC2373367

[B148] Stramer B. Wood W. Galko M. J. Redd M. J. Jacinto A. Parkhurst S. M. (2005). Live imaging of wound inflammation in *Drosophila* embryos reveals key roles for small GTPases during *in vivo* cell migration. J. Cell. Biol. 168, 567–573 10.1083/jcb.200405120 15699212 PMC2171743

[B149] Takehana A. Yano T. Mita S. Kotani A. Oshima Y. Kurata S. (2004). Peptidoglycan recognition protein (PGRP)-LE and PGRP-LC act synergistically in *Drosophila* immunity. EMBO J. 23, 4690–4700 10.1038/sj.emboj.7600466 15538387 PMC533052

[B150] Takekawa M. Saito H. (1998). A family of stress-inducible GADD45-like proteins mediate activation of the stress-responsive MTK1/MEKK4 MAPKKK. Cell 95, 521–30 10.1016/S0092-8674(00)81619-0 9827804

[B151] Tang H. Kambris Z. Lemaitre B. Hashimoto C. (2006). Two proteases defining a melanization cascade in the immune system of *Drosophila*. J. Biol. Chem. 281, 28097–28104 10.1074/jbc.M601642200 16861233

[B152] Tanigaki K. Honjo T. (2007). Regulation of lymphocyte development by Notch signaling. Nat. Immunol. 8, 451–456 10.1038/ni1453 17440450

[B153] Tepass U. Fessler L. I. Aziz a Hartenstein V. (1994). Embryonic origin of hemocytes and their relationship to cell death in *Drosophila*. Development 120, 1829–1837 7924990 10.1242/dev.120.7.1829

[B154] Theopold U. Schmidt O. Söderhäll K. Dushay M. S. (2004). Coagulation in arthropods: defence, wound closure and healing. Trends Immunol. 25, 289–294 10.1016/j.it.2004.03.004 15145318

[B155] Tipping M. Perrimon N. (2013). *Drosophila* as a model for context-dependent tumorigenesis. J. Cell. Physiol. 1–20 10.1002/jcp.24427 23836429 PMC4034382

[B157] Tothova Z. Kollipara R. Huntly B. J. Lee B. H. Castrillon D. H. Cullen D. E. (2007). FoxOs are critical mediators of hematopoietic stem cell resistance to physiologic oxidative stress. Cell 128, 325–339 10.1016/j.cell.2007.01.003 17254970

[B158] Tucker P. K. Evans I. R. Wood W. (2011). Ena drives invasive macrophage migration in *Drosophila* embryos. Dis. Model Mech. 4, 126–134 10.1242/dmm.005694 21045209 PMC3008967

[B159] Vainchenker W. Constantinescu S. N. (2013). JAK/STAT signaling in hematological malignancies. Oncogene 32, 2601–2613 10.1038/onc.2012.347 22869151

[B161] Valanne S. Wang J. H. Rämet M. (2011). The *Drosophila* Toll signaling pathway. J. Immunol. 186, 649–656 10.4049/jimmunol.1002302 21209287

[B162] Waltzer L. Gobert V. Osman D. Haenlin M. (2010). Transcription factor interplay during *Drosophila* haematopoiesis. Int. J. Dev. Biol. 54, 1107–1115 10.1387/ijdb.093054lw 20711988

[B163] Wang L. D. Wagers A. J. (2011). Dynamic niches in the origination and differentiation of haematopoietic stem cells. Nat. Rev. Mol. Cell. Biol. 12, 643–655 10.1038/nrm3184 21886187 PMC4040463

[B164] Wang Z. Wilhelmsson C. Hyrsl P. Loof T. G. Dobes P. Klupp M. (2010). Pathogen entrapment by transglutaminase–a conserved early innate immune mechanism. PLoS Pathog. 6:e1000763 10.1371/journal.ppat.1000763 20169185 PMC2820530

[B165] Watson F. L. Püttmann-Holgado R. Thomas F. Lamar D. L. Hughes M. Kondo M. (2005). Extensive diversity of Ig-superfamily proteins in the immune system of insects. Science 309, 1874–1878 10.1126/science.1116887 16109846

[B166] Wertheim B. Kraaijeveld A. R. Schuster E. Blanc E. Hopkins M. Pletcher S. D. (2005). Genome-wide gene expression in response to parasitoid attack in *Drosophila*. Genome Biol. 6, R94 10.1186/gb-2005-6-11-r94 16277749 PMC1297650

[B167] Williams M. J. Ando I. Hultmark D. (2005). *Drosophila melanogaster* Rac2 is necessary for a proper cellular immune response. Genes Cells 10, 813–823 10.1111/j.1365-2443.2005.00883.x 16098145

[B168] Williams M. J. Wiklund M.-L. Wikman S. Hultmark D. (2006). Rac1 signalling in *the Drosophila* larval cellular immune response. J. Cell. Sci. 119(Pt 10), 2015–2024 10.1242/jcs.02920 16621891

[B169] Wood W. Faria C. Jacinto A. (2006). Distinct mechanisms regulate hemocyte chemotaxis during development and wound healing in *Drosophila melanogaster*. J. Cell. Biol. 173, 405–416 10.1083/jcb.200508161 16651377 PMC2063841

[B170] Wood W. Jacinto A. (2007). *Drosophila melanogaster* embryonic haemocytes: masters of multitasking. Nat. Rev. Mol. Cell. Biol. 8, 542–551 10.1038/nrm2202 17565363

[B171] Wood W. Jacinto A. Grose R. Woolner S. Gale J. Wilson C. (2002). Wound healing recapitulates morphogenesis in *Drosophila* embryos. Nat. Cell. Biol. 4, 907–912 10.1038/ncb875 12402048

[B172] Wu S. C. Liao C. W. Pan R. L. Juang J. L. (2012). Infection-induced intestinal oxidative stress triggers organ-to-organ immunological communication in *Drosophila*. Cell Host Microbe 11, 410–417 10.1016/j.chom.2012.03.004 22520468

[B173] Wu Y. Brock A. R. Wang Y. Fujitani K. Ueda R. Galko M. J. (2009). A blood-borne PDGF/VEGF-like ligand initiates wound-induced epidermal cell migration in *Drosophila* larvae. Curr. Biol. 19, 1473–1477 10.1016/j.cub.2009.07.019 19646875 PMC2783944

[B174] Wu Y. Zhou B. P. (2009). Inflammation: a driving force speeds cancer metastasis. Cell Cycle 8, 3267–3273 10.4161/cc.8.20.9699 19770594 PMC3702728

[B175] Xavier M. J. Williams M. J. (2011). The Rho-family GTPase Rac1 regulates integrin localization in *Drosophila* immunosurveillance cells. PLoS ONE 6:e19504 10.1371/journal.pone.0019504 21603603 PMC3095607

[B176] Yang B. Lu A. Peng Q. Ling Q. Z. Ling E. (2013). Activity of fusion prophenoloxidase-GFP and its potential applications for innate immunity study. PLoS ONE 8:e64106 10.1371/journal.pone.0064106 23717543 PMC3662757

[B177] Zaidman-Rémy A. Regan J. C. Brandão A. S. Jacinto A. (2012). The *Drosophila* larva as a tool to study gut-associated macrophages: PI3K regulates a discrete hemocyte population at the proventriculus. Dev. Comp. Immunol. 36, 638–647 10.1016/j.dci.2011.10.013 22085781

[B178] Zhao C. Chen A. Jamieson C. H. Fereshteh M. Abrahamsson A. Blum J. (2009). Hedgehog signalling is essential for maintenance of cancer stem cells in myeloid leukaemia. Nature 458, 776–779 10.1038/nature07737 19169242 PMC2946231

